# A new class of PentixaFor- and PentixaTher-based theranostic agents with enhanced CXCR4-targeting efficiency

**DOI:** 10.7150/thno.45537

**Published:** 2020-07-09

**Authors:** Theresa Osl, Alexander Schmidt, Markus Schwaiger, Margret Schottelius, Hans-Jürgen Wester

**Affiliations:** 1 Chair for Pharmaceutical Radiochemistry, Faculties of Chemistry and Medicine, Technische Universität München, Garching, Germany.; 2 Department of Nuclear Medicine, Klinikum rechts der Isar, Technische Universität München, Munich, Germany.; 3 Translational Radiopharmaceutical Sciences, Departments of Nuclear Medicine and of Oncology, Centre Hospitalier Universitaire Vaudois and University of Lausanne, Lausanne, Switzerland.

**Keywords:** CXCR4, cyclic pentapeptide, PET, radioligand therapy, cancer

## Abstract

Non-invasive PET imaging of CXCR4 expression in cancer and inflammation as well as CXCR4-targeted radioligand therapy (RLT) have recently found their way into clinical research by the development of the theranostic agents [^68^Ga]PentixaFor (cyclo(D-Tyr^1^-D-[NMe]Orn^2^(AMBS-[^68^Ga]DOTA)-Arg^3^-Nal^4^-Gly^5^) = [^68^Ga]DOTA-AMBS-CPCR4) and [^177^Lu/^90^Y]PentixaTher (cyclo(D-3-iodo-Tyr^1^-D-[NMe]Orn^2^(AMBS-[^177^Lu/^90^Y]DOTA)-Arg^3^-Nal^4^-Gly^5^) = [^177^Lu/^90^Y]DOTA-AMBS-*iodo*CPCR4). Although convincing clinical results have already been obtained with both agents, this study was designed to further investigate the required structural elements for improved ligand-receptor interaction for both peptide cores (CPCR4 and *iodo*CPCR4). To this aim, a series of DOTA-conjugated CPCR4- and *iodo*CPCR4-based ligands with new linker structures, replacing the AMBA-linker in PentixaFor and PentixaTher, were synthesized and evaluated.

**Methods:** The *in vitro* investigation of the novel compounds alongside with the reference peptides PentixaFor and PentixaTher encompassed the determination of hCXCR4 and mCXCR4 affinity (IC_50_) of the respective ^nat^Ga-, ^nat^Lu-, ^nat^Y- and ^nat^Bi-complexes in Jurkat and Eμ-myc 1080 cells using [^125^I]FC-131 and [^125^I]CPCR4.3 as radioligands, respectively, as well as the evaluation of the internalization and externalization kinetics of selected ^68^Ga- and ^177^Lu-labeled compounds in hCXCR4-transfected Chem-1 cells. Comparative small animal PET imaging studies (1h p.i.) as well as *in vivo* biodistribution studies (1, 6 and 48h p.i.) were performed in Daudi (human B cell lymphoma) xenograft bearing CB17 SCID mice.

**Results:** Based on the affinity data and cellular uptake studies, [^68^Ga/^177^Lu]DOTA-r-a-ABA-CPCR4 and [^68^Ga/^177^Lu]DOTA-r-a-ABA-*iodo*CPCR4 (with r-a-ABA = D-Arg-D-Ala-4-aminobenzoyl-) were selected for further evaluation. Both analogs show app. 10-fold enhanced hCXCR4 affinity compared to the respective references [^68^Ga]PentixaFor and [^177^Lu]PentixaTher, four times higher cellular uptake in hCXCR4 expressing cells and improved cellular retention. Unfortunately, the improved *in vitro* binding and uptake characteristics of [^68^Ga]DOTA-r-a-ABA-CPCR4 and -*iodo*CPCR4 could not be recapitulated in initial PET imaging studies; both compounds showed similar uptake in the Daudi xenografts as [^68^Ga]PentixaFor, alongside with higher background accumulation, especially in the kidneys. However, the subsequent biodistribution studies performed for the corresponding ^177^Lu-labeled analogs revealed a clear superiority of [^177^Lu]DOTA-r-a-ABA-CPCR4 and [^177^Lu]DOTA-r-a-ABA-*iodo*CPCR4 over [^177^Lu]PentixaTher with respect to tumor uptake (18.3±3.7 and 17.2±2.0 %iD/g, respectively, at 1h p.i. vs 12.4±3.7%iD/g for [^177^Lu]PentixaTher) as well as activity retention in tumor up to 48h. Especially for [^177^Lu]DOTA-r-a-ABA-CPCR4 with its low background accumulation, tumor/organ ratios at 48h were 2- to 4-fold higher than those obtained for [^177^Lu]PentixaTher (except for kidney).

**Conclusions:** The in-depth evaluation of a series of novel CPCR4- and *iodo*CPCR4 analogs with modified linker structure has yielded reliable structure-activity relationships. It was generally observed that a) AMBA-by-ABA-substitution leads to enhanced ligand internalization, b) the extension of the ABA-linker by two additional amino acids (DOTA-Xaa_2_-Xaa_1_-ABA-) provides sufficient linker length to minimize the interaction of the [M^3+^]DOTA-chelate with the receptor, and that c) introduction of a cationic side chain (Xaa_2_) greatly enhances receptor affinity of the constructs, obliterating the necessity for Tyr^1^-iodination of the pentapeptide core to maintain high receptor affinity (such as in [^177^Lu]PentixaTher).

As a result, [^177^Lu]DOTA-r-a-ABA-CPCR4 has emerged from this study as a powerful second-generation therapeutic CXCR4 ligand with greatly improved targeting efficiency and tumor retention and will be further evaluated in preclinical and clinical CXCR4-targeted dosimetry and RLT studies.

## Introduction

In cancer, the interaction of the chemokine receptor 4 (CXCR4) with its cognate ligand CXCL12 is implicated in virtually all aspects of tumorigenesis, tumor progression and metastasis 1, 2. This includes attraction of CXCR4 expressing immune cells during premalignant chronic inflammation and malignant transformation 3, formation of a tumor-supporting niche by recruitment of bone marrow derived progenitor cells, fibroblasts and pro-tumorigenic immune cell subsets (MSDCs, Treg, M2 macrophages) 4, homing of CXCR4 overexpressing tumor cells, auto- and paracrine stimulation of tumor growth, vasculogenesis, invasion and distant metastasis as well as therapy resistance and immune evasion 5, 6.

**Figure 1:**
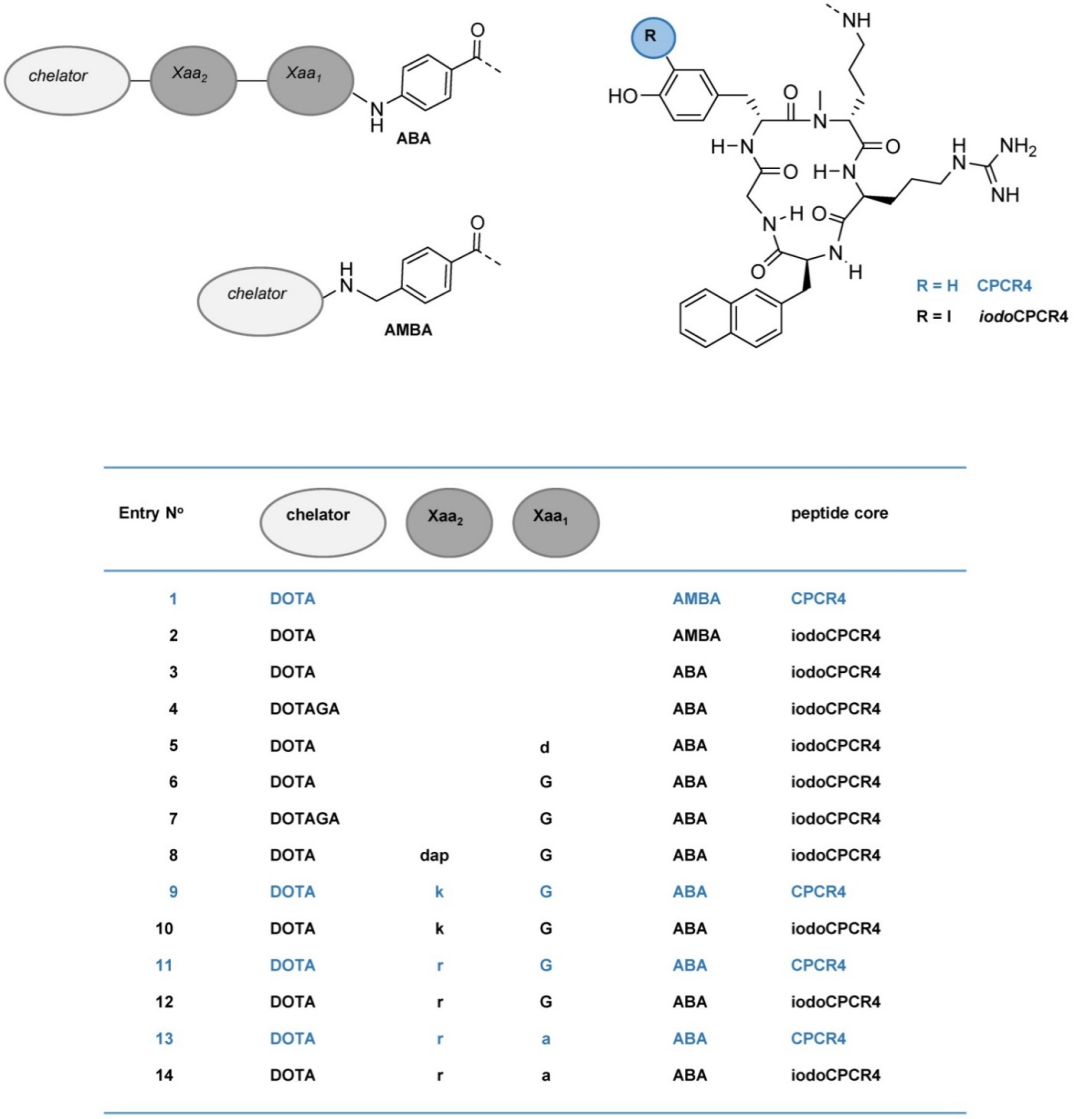
Schematic representation of the structures of the compounds investigated in this study, including PentixaFor (entry **1**), PentixaTher (entry **2**) and a series of CPCR4- (blue) and iodoCPCR4-(black) derived analogs with modified linker structure (entries **3**-**14**). For the amino acids in the linker unit (Xaa_1_ and Xaa_2_), one letter code was used for the sake of brevity. Abbreviations: DOTA: 2,2′,2”,2”'-(1,4,7,10-tetraazacyclododecane-1,4,7,10-tetrayl)tetraacetic acid; DOTAGA: 5-oxo-4-(4,7,10-tris(2-oxoethyl)-1,4,7,10-tetraazacyclododecan-1-yl) pentanoic acid; a: D-Ala; d: D-Asp; dap: D-2,3-diaminopropionic acid; G: Gly; k: D-Lys; r: D-Arg; ABA: 4-aminobenzoic acid; AMBA: 4-aminomethylbenzoic acid; CPCR4: cyclo(D-Tyr^1^-D-[NMe]Orn^2^(NH*)-Arg^3^-Nal^4^-Gly^5^); iodoCPCR4: cyclo(D-3-iodo-Tyr^1^-D-[NMe]Orn^2^(NH*)-Arg^3^-Nal^4^-Gly^5^), with * designating the attachment point for the linker, respectively.

These multiple facets make CXCR4 a valuable molecular marker with significant prognostic power 7 and a highly attractive target for non-invasive molecular imaging, triggering the development of a broad spectrum of CXCR4-targeted imaging probes during the last two decades 8, 9. Amongst these, only the cyclic pentapeptide [^68^Ga]PentixaFor (cyclo(D-Tyr^1^-D-[NMe]Orn^2^(AMBS-[^68^Ga]DOTA)-Arg^3^-Nal^4^-Gly^5^), [^68^Ga]DOTA-AMBS-CPCR4, Figure 1) 10-12 has found widespread application in the clinical setting so far 13, 14.

Its suitability for non-invasive high-contrast imaging of CXCR4 overexpressing cancers has been initially demonstrated for hematological malignancies 12, 15-18, but has also been successfully extended towards other tumor entities 19-27. By the subsequent development of [^177^Lu]PentixaTher (cyclo(D-3-iodo-Tyr^1^-D-[NMe]Orn^2^(AMBS-([^177^Lu]DOTA)-Arg^3^-Nal^4^-Gly^5^), [^177^Lu]DOTA-AMBS-*iodo*CPCR4, Figure 1) as a therapeutic companion 28, 29, a first CXCR4-targeted theranostic concept has recently been realized and translated into the clinic. After encouraging therapeutic responses using [^177^Lu/^90^Y] PentixaTher for radioligand therapy (RLT) of advanced disease in patients with multiple myeloma and other lymphoproliferative malignancies 30-32, the PentixaFor/PentixaTher-based theranostic concept is now being further explored by the license-holder PentixaPharm in prospective clinical trials.

In addition to its value for non-invasive detection of CXCR4 expressing tumor cells, [^68^Ga]PentixaFor-PET has also gained considerable attention in inflammation imaging, in particular in cardiovascular disease. After first pioneering studies showing specific [^68^Ga]PentixaFor uptake in macrophage-rich regions in atherosclerotic plaques in rabbits 33, several groups have investigated the suitability of [^68^Ga]PentixaFor-PET for clinical imaging of atherosclerosis 34-39, myocardial infarction 40-42, stroke 43 and other inflammatory conditions, i.e. infection 44, 45. In all these studies, [^68^Ga]PentixaFor PET was found to sensitively and reliably detect the CXCR4-expressing immune cell infiltration in areas of inflammation. More specifically, in atherosclerotic plaques, [^68^Ga]PentixaFor uptake was shown to be primarily mediated macrophages and to a lesser extent by T cells, as confirmed by immunohistochemistry 38.

Despite these encouraging clinical results, however, it is important to note that especially in the context of CXCR4+ immune cell imaging, sensitivity of [^68^Ga]PentixaFor PET leaves room for improvement. So far, achievable target/background ratios (TBR, e.g. in atherosclerosis) do not exceed 2.5-3 37, 38. This is sufficient for lesion detection; however, TBRs as well as general detection sensitivity for low-level CXCR4 expression may be substantially improved by using a CXCR4-targeted tracer with higher CXCR4 affinity than [^68^Ga]PentixaFor.

As described previously, [^68^Ga]PentixaFor interacts in its entirety with the binding pocket of CXCR4 46, leaving virtually no room for structural modifications without compromising receptor affinity. With the development of [^177^Lu]PentixaTher, i.e. the use of the *iodo*CPCR4 core peptide, receptor affinity was improved and at least some more flexibility towards changes in the chelator-M^3+^ complex geometry was gained 29. On the basis of these findings, we designed the present study to further explore the feasibility of modifying the respective PentixaFor (CPCR4-based) and PentixaTher (*iodo*CPCR4-based) parent peptides in a way that would yield compounds with:

- improved receptor affinity for more sensitive CXCR4 imaging, and;
- enhanced uptake and retention for therapeutic application.

To achieve this, we chose to primarily focus on optimizing the linker structure between the core peptides (CPCR4 and *iodo*CPCR4) and the radiolabel (M^3+^-DOTA). We hypothesized that this would not only provide a better understanding of the structural requirements for optimal interaction of the linker with the CXCR4 binding pocket, but might ultimately allow us to define the required linker length to reach outside of the binding site. This, i.e. the resulting prevention of direct interaction of the radiolabel itself with the receptor, would then in turn provide the basis for extending the CPCR4/*iodo*CPCR4-based CXCR4 targeting concept towards a variety of other radiolabeling strategies with fundamentally different structural requirements.

Thus, a series of second-generation analogs of PentixaFor and PentixaTher with modified linker structure (Figure 1) was synthesized and comparatively evaluated. By including both peptide scaffolds (CPCR4 and *iodo*CPCR4) in parallel into the study, we aimed at additionally assessing the relevance of D-Tyr^1^-iodination on the targeting behavior of the novel ligands. For a better placement of the results of this SAR study, we also included ^nat/68^Ga-PentixaFor and ^nat/177^Lu-PentixaTher as reference compounds into this study.

## Materials and Methods

### Synthesis and Radiolabeling

All peptides and their respective ^nat^Ga-, ^nat^Lu-,^ nat^Y- and ^nat^Bi- complexes were synthesized in analogy to previously published protocols 47-50. A detailed synthesis protocol, analytical data for the peptides investigated in this study and a description of the ^68^Ga- and ^177^Lu-labeling conditions are provided in the supplementary material. The radioiodinated reference ligands [^125^I]FC-131 and [^125^I]CPCR4.3 were prepared as described previously 51.

### Lipophilicity

The lipophilicity (log P_O/PBS_) of the ^68^Ga- and ^177^Lu-labeled compounds in this study was determined via a modified shake-flask method 52.

### *In vitro* evaluation

For *in vitro* experiments, the following cell lines were used: hCXCR4-expressing Jurkat human T-cell leukemia cells, Chemicon's Wild-Type (Chem-1) cells stably transfected with hCXCR4 and mCXCR4-expressing Eµ-Myc1080 mouse B-cell lymphoma cells 53. Jurkat cells were cultured in RPMI 1640 medium (Biochrom, Germany) containing 10% fetal calf serum (FCS) (Biochrom, Germany). Chem-1 cells were maintained in DMEM medium (Biochrom, Germany) supplemented with 10% FCS, 1% non-essential amino acids (Biochrom, Germany) and 1% HEPES (1M). Murine Eµ-Myc1080 cells were grown in RPMI 1640 medium supplemented with 20% FCS, 1% non-essential amino acids (Biochrom, Germany) and 0.1% 2-mercaptoethanol (Sigma-Aldrich, Germany). All cell lines were maintained at 37°C in a humidified atmosphere with 5% CO_2_.

### Determination of hCXCR4/mCXCR4 affinity

Competition binding studies (IC_50_) were performed as described 11 using either Jurkat cells (4×10^5^ cells/sample) and [^125^I]FC131 as radioligand or Eµ-Myc1080 mouse B-cell lymphoma cells (2×10^5^ cells/sample) and [^125^I]CPCR4.3 as radioligand 51. Experiments were performed in triplicate with n=3 per concentration in each experiment. IC_50_ values were calculated using GraphPad Prism 6.01 (Graph Pad Software, San Diego, USA).

### Internalization and externalization studies

Internalization kinetics of selected ^177^Lu-labeled compounds (1 nM) into Chem-1 cells were investigated in analogy to a previously published protocol 54. Non-specific internalization was determined in the presence of 10 µM AMD3100.

To determine ligand washout and recycling kinetics, Chem-1 cells were first incubated with the respective radioligand (1 nM) in assay medium (RPMI 1640, 5% BSA) for 120 min at 37 °C and then washed with HBSS. In the experiment allowing ligand recycling, 250 µL of assay medium were added to the wells (n = 3). In the experiment inhibiting ligand recycling, 250 µL of assay medium containing 10 µM AMD3100 were added to the wells (n = 3). Subsequently, cells were incubated at 37 °C for 5, 15, 30 and 60 min, respectively. The supernatant was removed and combined with 250 µL of HBSS used for rinsing the cells. This fraction represents the amount of externalized ligand at the respective time point. Then, cells were lysed using 300 µL of 1N NaOH. The lysate was transferred to vials and combined with 250 µL of HBSS used for rinsing the wells. Quantification of the amount of free, externalized and remaining cellular activity was performed using a Gamma Counter.

### *In vivo* experiments

The biodistribution of selected compounds was investigated in Daudi (human B-cell lymphoma) xenograft bearing female CB-17 SCID mice (6-8 weeks, Charles River, Sulzfeld, Germany) 12, 29. All animal experiments were performed in accordance with current animal welfare regulations in Germany (approval #55.2-1-54-2532-71-13).

Animals (n=4-5 per group) were injected intravenously with 5-10 MBq (0.08-0.11 nmol peptide per mouse) of the respective ^68^Ga- and^ 177^Lu-labeled CXCR4 ligands in PBS (100 µL) into the tail vein under isoflurane anesthesia. CXCR4-specificity of binding was determined by coinjection of 50 µg AMD3100 per mouse. The animals were sacrificed 1, 6 and 48h post injection (p.i.), and the organs of interest were dissected. The radioactivity was measured in weighted tissue samples using a γ-counter. Data are expressed in % ID/g tissue (mean ± SD).

Small aminal PET imaging studies were performed using a Siemens Inveon PET scanner. Animals were anesthetized with isoflurane and injected via the tail vein with 10 to 15 MBq (0.1 nmol) of the respective tracer. Dynamic imaging (n=1 per compound) was performed after on-bed injection for 1.5 h, and static images (n=1 per compound) were acquired at 1 h p.i. with an acquisition time of 15 min. Data analysis was performed using the Inveon Research Workplace software. Images were reconstructed using a 3D ordered-subsets expectation maximum (OSEM3D) algorithm without scanner and attenuation correction. Time-activity curves (Figure 4) were obtained by generating isocontour regions of interest (ROI) for the tumor and the heart content (as a surrogate for blood activity), as well as by defining two spherical ROIs (each 23.4 mm^3^) in the thigh area (muscle) and areas of homogenous tracer uptake in the liver, followed by plotting of average activity ± SD per volume in these ROIs over time.

### Statistical analysis

Statistical analysis (one-tailed t-test) of the biodistribution data sets was performed using Microsoft Excel.

## Results and Discussion

### Design of second-generation ligands, CXCR4 binding affinity and SAR study

**Figure 2:**
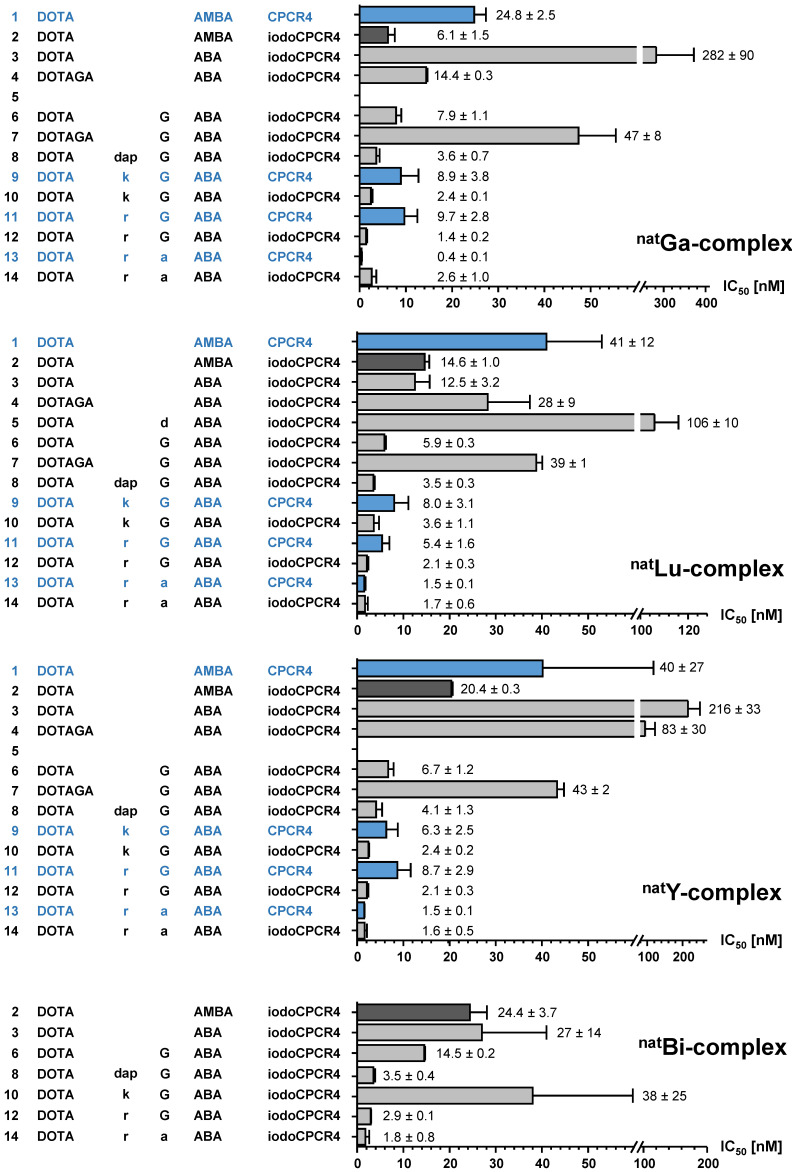
Binding affinities (IC_50_ in nM) of the respective ^nat^Ga-, ^nat^Lu-, ^nat^Y- and ^nat^Bi-complexes of PentixaFor (entry **1**), PentixaTher (entry **2**) and a series of CPCR4-(blue) and iodoCPCR4-(black) derived analogs with modified linker structure (entries **3**-**14**) to human CXCR4 (hCXCR4). Affinities to hCXCR4 were determined using Jurkat T-cell lymphoma cells (400.000 cells/sample) and [^125^I]FC-131 as radioligand. Each experiment was performed in triplicate, and results are means ± SD from a minimum of three separate experiments.

In the present series of second-generation CPCR4- and *iodo*CPCR4-based ligands, the AMBA (4-**a**mino**m**ethyl**b**enzoic **a**cid) moiety in PentixaFor and PentixaTher was first replaced by ABA (4-**a**mino**b**enzoic **a**cid); then, the linker was stepwise extended by selected additional amino acids. This and the alternative use of DOTAGA as bifunctional chelator led to the generation of the 12 novel peptide constructs summarized in Figures 1 and 2 (entries 3-14).

This design was based on important findings from a previous study 10, which had shown that:

- AMBA in ^nat^Ga-PentixaFor could be replaced by ABA or ABA-Gly without dramatically compromising CXCR4 affinity;
- Introduction of the ABA-Gly linker led to a twofold improvement of the affinity [^nat^In]DOTA-Gly-ABA-CPCR4 compared to [^nat^In]DOTA-AMBA-CPCR4 (=[^nat^In]DOTA-PentixaFor), and;
- All ABA-analogs exhibited a substantially enhanced ligand internalization compared to the respective AMBA-counterparts.

We thus recapitulated these modifications using the *iodo*CPCR4 scaffold to investigate the validity of our previous data for this modified peptide backbone (entries 2-7, Figure 2), and then expanded the concept by further extending the linker unit (entries 8, 10, 12 and 14, Figure 2). Of note, since the development of an optimized therapeutic analog was of particular interest to us in the context of this study, the most comprehensive *in vitro* data set has been acquired for the ^177^Lu/^nat^Lu-complexes of the novel CPCR4- and *iodo*CPCR4 derivatives (Figure 2). Consequently, in the following, their characteristics will be representatively discussed in most detail, and reference to the respective ^nat^Ga-, ^nat^Y- and ^nat^Bi-analogs will only be made for specific and noteworthy examples. For the sake of brevity, the one letter code is employed from here on in the compound names to describe the linker composition.

As mentioned, first structure-activity relationships (SAR) had already been obtained for [^nat^In]DOTA-AMBA-CPCR4 ([^nat^In]PentixaFor), [^nat^In]DOTA-ABA-CPCR4 and [^nat^In]DOTA-G-ABA-CPCR4 10. Overall, their CXCR4 affinities were by a factor of 2-10 lower than those of the corresponding ^nat^Ga-complexes, highlighting the impact of the M^3+^-DOTA-complex geometry on ligand interaction with the CXCR4 binding site. We had also observed in a separate study, that the latter effect could efficiently be counterbalanced by alternatively using *iodo*CPCR4 as the targeting peptide, as exemplified by [^nat^Lu]PentixaTher (entry 2, Figure 2) 29.

Thus, we first investigated whether the *iodo*CPCR4 backbone in conjunction with the ABA- (entry 3) and the G-ABA- (entry 6) linkers would also provide Lu^3+^-, Y^3+^- and/or Bi^3+^-complexed ligands with similar or even improved CXCR4 affinity compared to the respective ^nat^Ga counterparts. As shown in Figure 2, ABA-for-AMBA substitution (entry 3) is well tolerated for the respective Lu^3+^ and Bi^3+^ complexes, whereas it leads to a reduction in receptor affinity for the Ga^3+^ and Y^3+^ analogs. In contrast, the influence of the ABA-by-(G-ABA) substitution (entry 6) is more homogenous, generally entailing improved CXCR4 affinity, irrespective of the M^3+^ ion in the chelate. This was taken as a first indication that an extension of the linker unit (as compared to AMBA and ABA) might be beneficial with respect to increased tolerance of structural modifications “at the far end” of the tracer molecule, and thus, the G-ABA-linker was selected as the starting point for further optimizations.

To test this hypothesis, DOTA was replaced by DOTAGA in both DOTA-ABA-*iodo*CPCR4 and DOTA-G-ABA-*iodo*CPCR4, leading to entries 4 and 7 (Figure 2). For both [^nat^Lu]DOTAGA-ABA-*iodo*CPCR4 (entry 4) and [^nat^Lu]DOTAGA-G-ABA-*iodo*CPCR4 (entry 7), DOTAGA-conjugation lead to a marked decrease in CXCR4 affinity compared to their DOTA analogs (entries 3 and 6). The same was observed for the corresponding ^nat^Ga- and ^nat^Y complexes of DOTAGA-G-ABA-*iodo*CPCR4 (entry 7) vs DOTA-G-ABA-*iodo*CPCR4 (entry 6).

Interestingly, this modification was similarly deleterious to CXCR4 affinity as already observed for the DOTAGA-analogs of PentixaFor, i.e. [^nat^Ga/^ nat^Lu/^ nat^Y]DOTAGA-AMBA-CPCR4, in a previous study 48. Therefore, it was concluded that a further extension of the linker was necessary to annihilate the observed effect.

Since obviously the introduction of a negative charge into the linker unit, as exemplified by [^nat^Lu]DOTA-d-ABA-*iodo*CPCR4 (entry 5), had negative impact on the binding affinity, a selection of different cationic amino acids (D-2,3-diaminopropionic acid (dap), D-Lys (k) and D-Arg (r)) was alternatively introduced to further extend the G-ABA-linker (entries 8, 10 and 12). The choice of cationic amino acids in this position was based on docking data obtained by co-crystallization of CXCR4 and CVX5, a 16-amino acid CXCR4 antagonist, which had revealed a salt bridge between the Lys^7^-residue of the peptide and D193 of the receptor 55. This salt bridge is positioned at the entrance to the actual binding pocket, with the pharmacophore being deeply embedded in the pocket and the β-turn region of CVX5 pointing towards the exterior of the binding pocket. Thus, we hypothesized that extension of the G-ABA-linker by a positively charged amino acid might at the same time provide additional strong binding interaction via the salt bridge and provide the necessary linker length for placing any N-terminal modification of the linker (M^3+^-DOTA or other) outside the binding site, thereby minimizing the effect of structural changes in this position on CXCR4 affinity.

As summarized in Figure 2, the introduction of D-Dap (dap) into the linker unit (entry 8) and its sequential substitution by D-Lys (k, entry 10) and D-Arg (r, entry 12) indeed led to the intended effect, i.e. progressively increasing CXCR4 affinities of the respective ligands. Most notably, this effect was independent from the nature of the M^3+^-DOTA-complex, yielding equal and very high receptor affinities for [^nat^Ga/^ nat^Lu/^ nat^Y/^ nat^Bi]DOTA-r-G-ABA-*iodo*CPCR4. Both these observations confirm the validity of the hypotheses outlined above.

In a last step, taking into consideration the beneficial effects of peptide stabilization (conformational as well as metabolic) via Gly-by-D-Ala substitution, the linker in the so far most affine peptide, DOTA-r-G-ABA-*iodo*CPCR4 (entry 12) was modified accordingly, yielding DOTA-r-a-ABA-*iodo*CPCR4 (entry 14). Again, this modification provided unchanged or even enhanced CXCR4 affinity for all M^3+^-DOTA-complexes compared to the respective Gly-counterparts (entry 12), alongside with an anticipated superior *in vivo* stability towards degradation by peptidases.

So far, however, all successive linker optimizations had been performed using the *iodo*CPCR4 scaffold, which had been shown to entail an improved binding affinity and tolerance towards structural modifications at the linker site by itself. It was thus of considerable interest to evaluate, if the optimized linker(s) (entries 10, 12 and 14) alone would be sufficient to also convey the desired properties, i.e. improved CXCR4 affinity combined with flexibility towards modification at far end of the linker, to ligands based on the unmodified CPCR4 backbone.

Quite interestingly, and again irrespective of the nature of the metal ion in the DOTA chelate, the CXCR4 affinity of DOTA-k-G-ABA-CPCR4 (entry 9) and DOTA-r-G-ABA-CPCR4 (entry 11) was still markedly lower than that of their respective *iodo*CPCR4 counterparts (entries 10 and 12). However, in the case of the DOTA-r-a-ABA-linker constructs (entries 13 vs 14), these differences were not observable any more. This finding suggests that ligand interaction with the CXCR4 binding pocket is at this point also determined by the strong and highly optimized binding interaction of the r-a-ABA linker itself with the receptor protein, partly overriding the influence of the different peptide scaffolds.

Since however, Tyr^1^-iodination of PentixaFor has been shown to exert considerable effects on tracer characteristics other than affinity, i.e. lipophilicity and clearance characteristics 29, and might thus display quite different *in vivo* performance, both DOTA-r-a-ABA-CPCR4 (entry 13) and DOTA-r-a-ABA-*iodo*CPCR4 (entry 14) were chosen as lead candidates for further evaluation, labeled both with ^68^Ga for PET imaging and ^177^Lu for potential therapeutic application.

For the appropriate interpretation of preclinical biodistribution and imaging studies in mouse models such as the Daudi human B-cell lymphoma xenograft model in this study, however, knowledge of the species dependence of target binding is of utmost importance. Consequently, the affinity of the ^177^Lu-complexes of selected *iodo*CPCR4-analogs with optimized linker structure to mouse CXCR4 (mCXCR4) was also determined. Since it had previously been shown that tracers based on the CPCR4 scaffold generally have substantially lower affinity to mCXCR4 than their *iodo*CPCR4 counterparts 29, only [^177^Lu]DOTA-r-a-ABA-CPCR4 was included in the comparative affinity determination.

As summarized in Table 1, the changes in mCXCR4 affinity associated with the consecutive linker optimizations (k-G-ABA to r-G-ABA to r-a-ABA) are quite similar to those observed for hCXCR4 (Figure 2). They also indicate an advantage for D-Arg over D-Lys in the linker, leading to improved mCXCR4 affinity. In contrast, Gly-by-D-Ala substitution entails a slightly decreased mCXCR4 affinity for [^177^Lu]DOTA-r-a-ABA-*iodo*CPCR4 compared to the r-G-ABA-derivative. As expected, the mCXCR4 affinity of [^177^Lu]DOTA-r-a-ABA-CPCR4 is substantially lower than that of the *iodo*CPCR4-analogs, highlighting once more the relevance of this Tyr^1^-iodination for efficient ligand interaction with the murine receptor.

### Internalization and externalization studies

**Figure 3:**
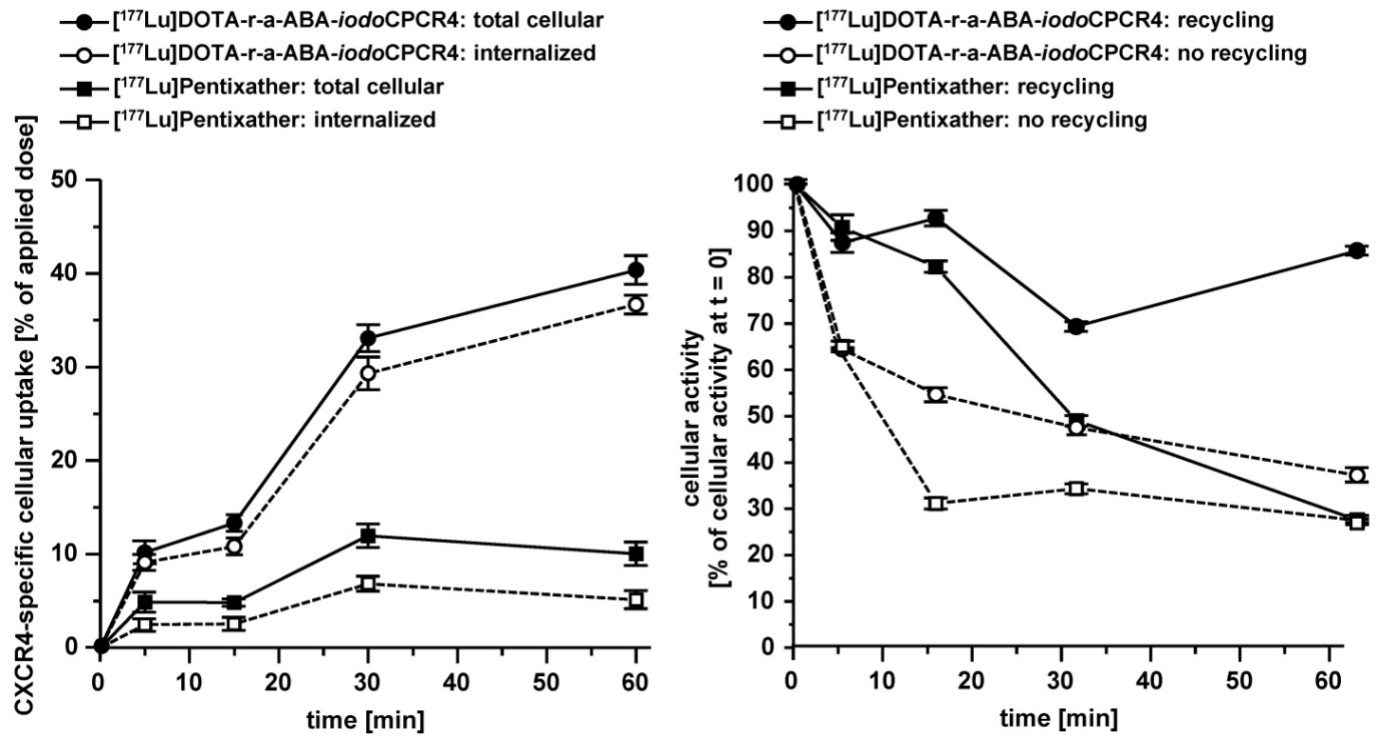
Left panel: comparative total cellular uptake and internalization kinetics for [^177^Lu]DOTA-r-a-ABA-iodoCPCR4 and [^177^Lu]PentixaTher. Right panel: comparative externalization kinetics for [^177^Lu]DOTA-r-a-ABA-iodoCPCR4 and [^177^Lu]PentixaTher (after a 120 min pre-incubation to allow tracer uptake) under conditions allowing (medium only) or inhibiting (10 μM AMD3100in external medium) tracer reinternalization (recycling). Experiments were performed at 37°C using Chem-1 cells stably transfected with hCXCR4. Each experiment was performed in triplicate, and results are means ± SD.

In a next step, to complement the previous structure-affinity-relationships by functional information, the internalization and externalization kinetics selected CPCR4/*iodo*CPCR4 analogs were also investigated (Table 2, Figure 3). Again, studies were focused on the respective ^177^Lu-labeled tracers due to the particular relevance of their internalization and retention characteristics for targeted RLT (Table 2, Figure 3). Besides the reference [^177^Lu]PentixaTher, [^177^Lu]DOTA-ABA-*iodo*CPCR4 was evaluated to be able to specifically assess the influence of the AMBA-to-ABA transition on cellular tracer uptake; furthermore, the respective complementary [^177^Lu]DOTA-r-G-ABA-CPCR4/*iodo*CPCR4 and [^177^Lu]DOTA-r-a-ABA-CPCR4/*iodo*CPCR4 pairs were also included to evaluate the relevance of the alternative peptides core vs the respective linker structure for efficient tracer uptake.

Interestingly, despite nearly identical receptor affinities, [^177^Lu]PentixaTher and [^177^Lu]DOTA-ABA-*iodo*CPCR4 showed very distinct internalization behavior, with the ABA-analog displaying a 70% increase in total cellular uptake, with a substantially higher fraction of this activity being internalized than for [^177^Lu]PentixaTher. This finding confirmed our previous preliminary result, that the ABA linker itself already conveys enhanced internalization efficiency to CPCR4-based radioligands. However, this effect is further enhanced by the introduction of the additional linker extension via the r-G- and r-a-moieties, respectively. As summarized in Table 2, both [^177^Lu]DOTA-r-G-ABA-*iodo*CPCR4 and [^177^Lu]DOTA-r-a-ABA-*iodo*CPCR4 show fourfold higher total cellular uptake than [^177^Lu]PentixaTher (also see Figure 3), and for both compounds, more than 90% of the cellular activity were found to be internalized, respectively. While the increased total cellular uptake of these compounds was not unexpected, given their improved hCXCR4 affinity, the enhanced internalization efficiency does not seem to be a function of receptor affinity, as exemplified by the data for the two analogous CPCR4-counterparts, [^177^Lu]DOTA-r-G-ABA-CPCR4 and [^177^Lu]DOTA-r-a-ABA-CPCR4. For these two compounds, total cellular uptake again correlates well with their respective hCXCR4 affinities (Figure 2), but their internalization efficiency remained almost unchanged compared to [^177^Lu]PentixaTher.

Based on these substantial differences in internalization profile between the respective CPCR4- and *iodo*CPCR4-analogs, we hypothesized that combining the new optimized linker structure with the *iodo*CPCR4 backbone might have led to a shift from an antagonistic towards an agonistic ligand profile. This assumption is supported by data from the literature, describing an identical divergence in cellular uptake characteristics between e.g. somatostatin or GRP-receptor targeted agonistic and antagonistic radioligands 57, 58. While the agonists generally displayed efficient ligand internalization, leading to high intracellular activity accumulation, the (oftentimes enhanced) cellular uptake of the corresponding receptor antagonists was found to primarily be due to ligand binding to receptor molecules on the cell surface, with negligible intracellular activity accumulation. The particularly high absolute cellular uptake of the antagonists despite a lack of endocytosis was explained by an increased number of available binding sites (including inactivated receptors) for antagonist binding.

To confirm the assumption that the observed uptake characteristics for [^177^Lu]DOTA-r-a-ABA-*iodo*CPCR4 as compared to [^177^Lu]PentixaTher (and [^177^Lu]DOTA-r-a-ABA-CPCR4) were indeed the result of agonistic activity, a cAMP assay was performed (Suppl. Material). Indeed, while [^nat^Lu]PentixaTher inhibited intracellular cAMP degradation induced by CXCL12-mediated G_i_ signaling in a concentration dependent manner, clearly confirming its antagonistic properties, [^nat^Lu]DOTA-r-a-ABA-*iodo*CPCR4 was found to further potentiate CXCL12 mediated signaling (Suppl. Figure 1) and was thus classified as a weak partial agonist of CXCR4. Although not specifically investigated for the corresponding CPCR4-counterpart [^nat^Lu]DOTA-r-a-ABA-CPCR4, the cellular uptake characteristics of this peptide rather suggest an antagonistic nature for this peptide (Table 2). These distinct features can be anticipated to have significant impact on *in vivo* CXCR4 targeting, and thus, [^68^Ga]DOTA-r-a-ABA-CPCR4/*iodo*CPCR4 and [^177^Lu]DOTA-r-a-ABA-CPCR4/*iodo*CPCR4 were selected as complementary ligand pairs for *in vivo* comparison with the references [^68^Ga]PentixaFor and [^177^Lu]PentixaTher, respectively.

This selection was also supported by the subsequent investigation of the cellular retention properties of the ^177^Lu-labeled compounds selected for functional *in vitro* evaluation (Table 2 and Figure 3). Fast and high tracer uptake as well as prolonged activity retention in the tumor are key components for achieving high dose rates to the tumor during RLT, and both features were observed for [^177^Lu]DOTA-r-a-ABA-CPCR4 and [^177^Lu]DOTA-r-a-ABA-*iodo*CPCR4.

Interestingly, when ligand recycling, i.e. reuptake of externalized ligand, was prohibited by an excess of unlabeled competitor (100 μM AMD3100), all compounds investigated showed almost identical and comparably low cellular retention. Thus, ligand structure obviously has no noteworthy influence on their intracellular handling and externalization kinetics; at the same time, their highly similar retention/release profile indicates a comparable *in vitro* stability for all CPCR4 and *iodo*CPCR4 analogs investigated.

However, significant differences between tracer groups become apparent in the experiment allowing ligand-reuptake after externalization (Table 2 and Figure 3). While for [^177^Lu]PentixaTher and [^177^Lu]DOTA-ABA-*iodo*CPCR4, recycling only provides for a slight increase in apparent tracer retention, the effect is much more pronounced for all analogs with the optimized linker structures. Especially for the DOTA-r-a-ABA-derivatives, an apparent activity retention of >85% is observed. If this apparent retention is occasioned by efficient re-binding of the ligand to the receptor (as may be hypothesized for the supposed antagonist [^177^Lu]DOTA-r-a-ABA-CPCR4) or by rapid reinternalization (for the agonist [^177^Lu]DOTA-r-a-ABA-*iodo*CPCR4), however, has not been elucidated in detail. Nevertheless, their observed particularly high apparent cellular retention additionally confirms their previous selection as most promising candidates for further *in vivo* evaluation.

### Determination of lipophilicity

Since lipophilicity is one of the key determinants for the general biodistribution and excretion pattern of peptide radiopharmaceuticals, a comparative logP_O/W_ determination was also performed, including both the ^68^Ga- and the ^177^Lu-labelled variants for the new analogs investigated (Table 3).

All findings correlated well with the anticipated effects of the introduction of the cationic linkers (r-G-ABA and r-a-ABA): generally, a reduction of tracer lipophilicity compared to the reference ligands was observed, with the beneficiary effect being substantially more pronounced in the case of the *iodo*CPCR4 analogs vs [^177^Lu]PentixaTher than for the CPCR4-derivative vs [^68^Ga]PentixaFor. Furthermore, all ^68^Ga-labeled compounds showed enhanced hydrophilicity compared to their ^177^Lu-labeled counterparts. This effect was also expected because of well-documented differences in complex geometry between the ^68^Ga- and the ^177^Lu-DOTA-monoamide complexes (as in peptide conjugates); in contrast to the octacoordinated Lu^3+^ ion, which utilizes oxygen donor atoms from all four pendant arms (three carboxylates and one carboxamide arm) of DOTA for complex stabilization, the carboxylate arm in trans position to the carboxamide arm remains uncoordinated in the hexacoordinate Ga^3+^-DOTA-complex 59, entailing additional polarity of the complex and thus enhanced hydrophilicity of the [^68^Ga]DOTA-conjugated peptides.

Interestingly, the marked increase in lipophilicity that had been induced by D-Tyr^1^-iodination in the case of the AMBA-derivatives [^177^Lu]PentixaTher vs [^68^Ga]PentixaFor is much less pronounced for the respective [^68^Ga/^177^Lu]DOTA-r-a-ABA-*iodo*CPCR4 vs -CPCR4 pairs, indicating the dominant influence of the positively charged linker unit on overall lipophilicity. Another interesting finding was the - unexpected - superior hydrophilicity of the [^68^Ga/^177^Lu]DOTA-r-a-ABA-*iodo*CPCR4 analogs compared their corresponding r-G-ABA counterparts, making them - again - the candidates with the most promising characteristics for *in vivo* evaluation.

### Small-animal PET imaging

**Figure 4:**
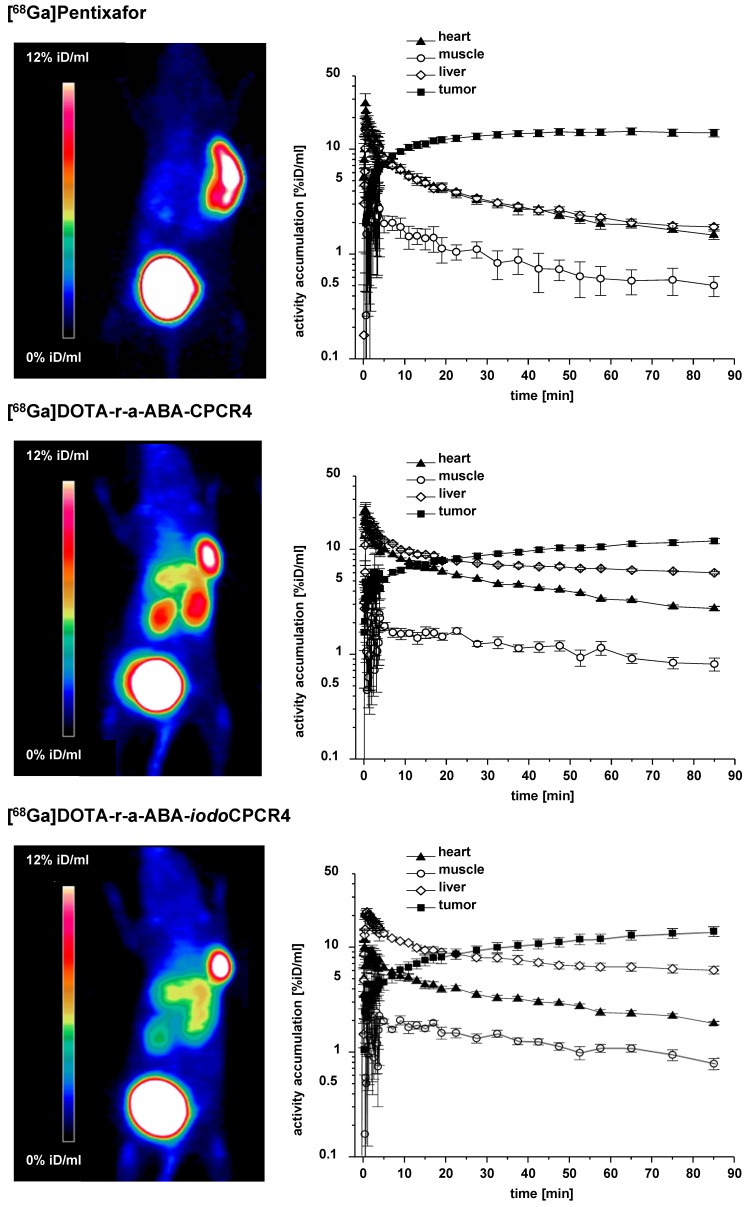
Results from small animal PET imaging using [^68^Ga]PentixaFor (top row), [^68^Ga]DOTA-r-a-ABA-CPCR4 (middle row) and [^68^Ga]DOTA-r-a-ABA-iodoCPCR4 (bottom row) in Daudi xenograft bearing CB17 SCID mice. Images on the left (MIP) were obtained by static imaging (1h p.i., n=1), the time activity curves (TAC) on the right were acquired by dynamic PET imaging (0-90 min p.i., n=1).

To get a first impression of the *in vivo* characteristics of the novel [^68^Ga]DOTA-r-a-ABA-analogs, comparative small animal PET imaging studies, including [^68^Ga]PentixaFor as a reference, were performed (Figure 4). To validate the results of the ROI analysis, the tissue distribution of [^68^Ga]DOTA-r-a-ABA-CPCR4 and -*iodo*CPCR4 (1h p.i.) was additionally investigated in a biodistribution study, including a blocking experiment to confirm CXCR4 specificity of tumor uptake (1h p.i., Supplementary Table S1 and Supplementary Figure S2). Quite surprisingly, and despite their outstanding hCXCR4 affinities, improved cellular uptake kinetics as well as reduced lipophilicities, both [^68^Ga]DOTA-r-a-ABA-CPCR4 and [^68^Ga]DOTA-r-a-ABA-*iodo*CPCR4 showed inferior imaging performance compared to [^68^Ga]PentixaFor.

This primarily due to enhanced background accumulation of the two novel r-a-ABA-analogs: firstly, both compounds show delayed blood clearance compared to the reference (see TACs in Figure 4), leading to higher background activity levels, as exemplified by the TAC for muscle. Secondly, quite in contradiction to their enhanced hydrophilicities, which were expected to further promote exclusive renal excretion, both [^68^Ga]DOTA-r-a-ABA-CPCR4 and [^68^Ga]DOTA-r-a-ABA-*iodo*CPCR4 both show 3-fold higher liver uptake (6.0±0.2 and 6.1±0.1 %iD/ml at 90 min p.i., respectively) compared to [^68^Ga]PentixaFor (1.8±0.1 %iD/ml). This was also confirmed in the corresponding biodistribution studies at 1h p.i. (Table S1). As depicted in the TACs in Figure 4, however, this increase in hepatic accumulation does not seem to be the consequence of enhanced hepatobiliary excretion, which would lead to steadily decreasing liver activities over time. On the contrary, both [^68^Ga]DOTA-r-a-ABA-CPCR4 and [^68^Ga]DOTA-r-a-ABA-*iodo*CPCR4 show pronounced hepatic retention over the observation period, whereas [^68^Ga]PentixaFor is efficiently cleared from the liver, with kinetics closely paralleling those of blood clearance.

One potential reason for this observation might lie in the enhanced mCXCR4 affinity of the novel a-r-ABA-constructs; as demonstrated previously, enhanced and sometimes even dramatic (> 40% iD/g) liver accumulation of CXCR4-targeted tracers in mice is always observed for ligands with affinity for mCXCR4 56, 60. The finding that this uptake is (at least partially) blockable by an excess of unlabeled competitor such as AMD3100 (see Table S1) suggests involvement of a specific uptake mechanism for CXCR4-targeted tracers in the mouse liver based on hepatic mCXCR4 expression 61.

Besides unfavorably high liver activity levels, both novel [^68^Ga]DOTA-a-r-ABA-analogs additionally display increased kidney uptake compared to the standard [^68^Ga]PentixaFor. With 4.3±0.4 and 9.4±0.2 %iD/ml at 90 min p.i. (data not shown in TACs), respectively, kidney uptake of [^68^Ga]DOTA-r-a-ABA-*iodo*CPCR4 and [^68^Ga]DOTA-r-a-ABA-CPCR4 exceeds that of [^68^Ga]PentixaFor (2.7±0.1 %iD/ml) by a factor of 2-4. This finding, however, which was also confirmed in the biodistribution study (Table S1), is not entirely unexpected, since both [^68^Ga]DOTA-r-a-ABA-constructs bear an additional positive charge in the linker unit, and it is well known that positively charged radiopeptides are preferentially accumulated in the kidney cortex by tubular reabsorption via the megalin/cubilin complex 62, 63.

Thus, overall, the introduction of the alternative r-a-ABA-linker structure has, despite favorably contributing to ligand hydrophilicity, undesired side effects on the overall biodistribution pattern of [^68^Ga]DOTA-r-a-ABA-CPCR4 and [^68^Ga]DOTA-r-a-ABA-*iodo*CPCR4 in comparison [^68^Ga]PentixaFor. At the same time, the small animal PET imaging as well as the biodistribution data (Table S1) show, that the 10- to 60-fold higher CXCR4 affinity of [^68^Ga]DOTA-r-a-ABA-CPCR4 and [^68^Ga]DOTA-r-a-ABA-*iodo*CPCR4 (Figure 2) is not reflected by enhanced tumor uptake of the novel analogs compared to the reference.

At 90 min p.i., all three ^68^Ga-labeled CXCR4 ligands show comparably high tumor accumulation in PET (14.3±1.2, 12.0±0.7 and 14.2±1.5 %iD/ml for [^68^Ga]PentixaFor, [^68^Ga]DOTA-r-a-ABA-CPCR4 and [^68^Ga]DOTA-r-a-ABA-*iodo*CPCR4, respectively; Figure 4). The corresponding biodistribution data at 1h p.i. (Supplemental Table S1) reveal similar (11.7±1.3 %iD/g for [^68^Ga]DOTA-r-a-ABA-CPCR4) or even lower (8.3±1.3 %iD/g for [^68^Ga]DOTA-r-a-*iodo*ABA-CPCR4) tumor accumulation for the novel linker conjugates. Thus, the anticipated differences between agonistic and antagonistic behavior, as discussed in the previous section, is not discernible. The only obvious difference in the tumor accumulation between the agonistic ligand [^68^Ga]DOTA-r-a-ABA-*iodo*CPCR4 and the antagonist [^68^Ga]PentixaFor is revealed by the respective TACs for tumor: while uptake of [^68^Ga]PentixaFor is fast and plateaus at app. 20 min p.i., the activity concentration of [^68^Ga]DOTA-r-a-ABA-*iodo*CPCR4 in tumor is steadily increasing over the entire observation period, supporting the notion of continuous ligand internalization. This feature, alongside with the delayed blood clearance observed for [^68^Ga]DOTA-r-a-ABA-*iodo*CPCR4, may represent a significant advantage for more efficient tumor targeting in a therapeutic setting. Thus, the ^177^Lu-labeled analog of DOTA-r-a-ABA-*iodo*CPCR4 was further evaluated in comparative biodistribution studies, also including [^177^Lu]DOTA-r-a-ABA-CPCR4 and the reference [^177^Lu]PentixaTher.

With respect to PET imaging, however, it must be concluded, that despite their undisputed advantages in *in vitro* CXCR4 targeting, both [^68^Ga]DOTA-r-a-ABA-CPCR4 and [^68^Ga]DOTA-r-a-ABA-*iodo*CPCR4 are not able to surpass or even match the imaging performance of [^68^Ga]PentixaFor.

### Biodistribution studies

**Figure 5:**
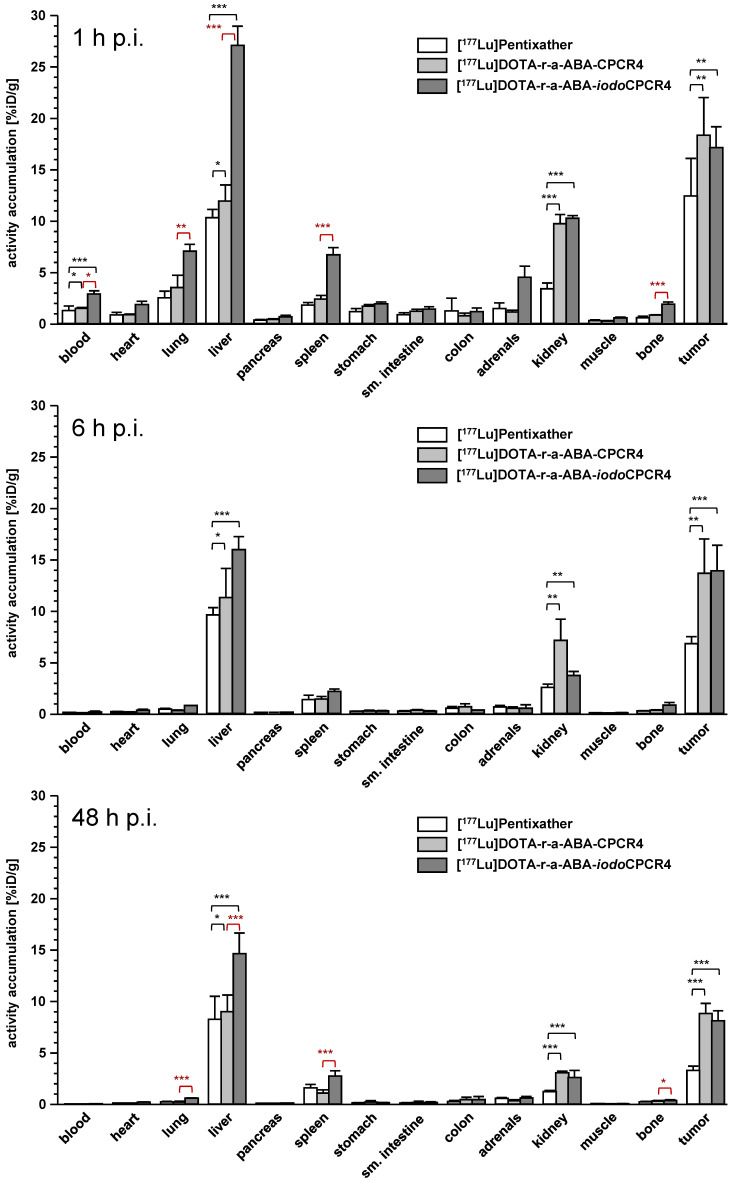
Biodistribution of [^177^Lu]PentixaTher, [^177^Lu]DOTA-r-a-ABA-CPCR4 and [^177^Lu]DOTA-r-a-ABA-iodoCPCR4 in Daudi xenograft bearing CB17 SCID mice at 1h (top), 6h (middle) and 48h p.i. (bottom). Data are given in %iD/g and are means ± SD (groups of n=4-5 animals).

In contrast, [^177^Lu]DOTA-r-a-ABA-CPCR4 and [^177^Lu]DOTA-r-a-ABA-*iodo*CPCR4, when compared to the corresponding therapeutic reference [^177^Lu]PentixaTher, do show superior tumor accumulation and retention up to 48h p.i. (Figure 5, supplementary tables S2-S4). Both novel analogs show up to 50% enhanced tumor uptake at early time points (18.3±3.7 and 17.2±2.0 %iD/g for [^177^Lu]DOTA-r-a-ABA-CPCR4 and [^177^Lu]DOTA-r-a-ABA-*iodo*CPCR4, respectively, vs 12.4±2.7 %iD/g for [^177^Lu]PentixaTher). As shown for its ^68^Ga-labeled counterpart (Table S1), tumor accumulation of [^177^Lu]DOTA-r-a-ABA-CPCR4 was confirmed to by highly CXCR4-specific (Table S2). Notably, [^177^Lu]DOTA-r-a-ABA-CPCR4 and [^177^Lu]DOTA-r-a-ABA-*iodo*CPCR4 also show a higher degree of activity retention after 48h. While app. 50% of the initial activity are retained in the Daudi xenografts after 48h for both novel linker conjugates, this fraction amounts to only 25% for [^177^Lu]PentixaTher.

In contrast to the ^68^Ga-labeled compounds, where the *in vivo* tumor uptake did not reflect their *in vitro* CXCR4 targeting behavior, a good correlation between *in vitro* and *in vivo* data was observed for the ^177^Lu-labeled analogs: firstly, the enhanced tumor accumulation of the two [^177^Lu]DOTA-r-a-ABA conjugates correlates well with their improved hCXCR4 affinity (Figure 2); secondly, their more persistent retention in tumor compared to [^177^Lu]PentixaTher reflects an improved cellular retention, as observed in the *in vitro* externalization studies (Table 2); and thirdly, slightly altered tumor uptake kinetics, as exemplified by the TACs for tumor of [^68^Ga]DOTA-r-a-ABA-*iodo*CPCR4 vs [^68^Ga]PentixaFor (Figure 4) and as discussed in the previous section, may also contribute to the observed effect.

Interestingly, however, and as already observed for the respective ^68^Ga-labeled analogs, there was no detectable difference between the tumor uptake and retention characteristics of [^177^Lu]DOTA-r-a-ABA-CPCR4 and [^177^Lu]DOTA-r-a-ABA-*iodo*CPCR4, despite obvious differences in their cellular uptake characteristics (Table 2 and Figure 5). Overall, both r-a-ABA-constructs showed improved tumor/organ ratios compared to [^177^Lu]PentixaTher (Table 4), with the effect being particularly pronounced for [^177^Lu]DOTA-r-a-ABA-CPCR4.

One the one hand, this is primarily the consequence of its enhanced tumor uptake, but is, on the other hand, further supported by the essentially unchanged background accumulation of [^177^Lu]DOTA-r-a-ABA-CPCR4 in all organs compared to [^177^Lu]PentixaTher except kidney (Figure 5, supplemental tables S2-S4). Not surprisingly, renal tracer uptake is substantially increased for the two r-a-ABA linker conjugates, most probably due to the increased overall number of positive charges in the tracer molecule, which has been shown to lead to increased peptide (re)absorption by the megalin-cubilin complex in the kidney 62.

In the case of [^177^Lu]DOTA-r-a-ABA-*iodo*CPCR4, the high tracer uptake in tumor is (partly) counterbalanced by the expected (see Figure 4) effect of Tyr^1^-iodination on general tracer pharmacokinetics, i.e. delayed blood clearance and resulting enhanced background accumulation as a result of slightly enhanced lipophilicity (Table 3), leading to lower tumor/background ratios than for the CPCR4-analog. Nevertheless, tumor/organ ratios of [^177^Lu]DOTA-r-a-ABA-*iodo*CPCR4 are still superior to those observed for [^177^Lu]PentixaTher.

It is important to note at this point, however, that due to its enhanced affinity towards mCXCR4, the biodistribution of [^177^Lu]DOTA-r-a-ABA-*iodo*CPCR4 is “biased” with respect to background accumulation in comparison to the analogs with significantly lower mCXCR4 binding affinity, [^177^Lu]PentixaTher and [^177^Lu]DOTA-r-a-ABA-CPCR4 (Table 1 and Figure 5). It has been shown for other radioligands with high mCXCR4 affinity, that tracer uptake in liver, spleen, lung and bone (femur harboring bone marrow) is blockable with an excess of cold competitor 56, 60. In the case of [^177^Lu]DOTA-r-a-ABA-*iodo*CPCR4, a blocking study has not been performed. However, we observed significantly enhanced accumulation of [^177^Lu]DOTA-r-a-ABA-*iodo*CPCR4 in mCXCR4-expressing tissues such as lung, liver, spleen and bone (Figure 5, statistical significance indicated in red) compared to [^177^Lu]DOTA-r-a-ABA-CPCR4. This observation cannot be satisfactorily explained by pharmacokinetic effects due to the (only slightly) different lipophilicities of the two compounds (Table 3), but strongly hints towards a significant contribution of mCXCR4-mediated uptake of [^177^Lu]DOTA-r-a-ABA-*iodo*CPCR4 in these tissues. This aspect must be taken into consideration when comparing the respective biodistribution patterns and tumor/background ratios for [^177^Lu]DOTA-r-a-ABA-*iodo*CPCR4 vs [^177^Lu]DOTA-r-a-ABA-CPCR4 and [^177^Lu]PentixaTher.

To exclude, that divergent *in vivo* stabilities may also be a factor contributing to the observed differences *in vivo* data obtained for [^177^Lu]DOTA-r-a-ABA-CPCR4 and [^177^Lu]DOTA-r-a-ABA-*iodo*CPCR4, an *in vivo* metabolite analysis was performed for both tracers (see Supplementary Material, Figure S3). Both compounds were found to be >99% stable in blood, urine and liver homogenates of CB17 SCID mice (Supp. Figure 2) at 0.5h p.i., suggesting minimal influence of tracer metabolism on initial biodistribution and tumor targeting. Of note, *in vivo* deiodination of 3-iodo-Tyr^1^, which is the most probable metabolic transformation anticipated for *iodo*CPCR4-based tracers, has not been observed in the observation window. Even if it were to occur at later time points, this metabolic step, in the case of the key compounds of the present study, would lead to the transformation of one potent CXCR4-targeted tracer into another at later time points, i.e. from [^177^Lu]DOTA-r-a-ABA-*iodo*CPCR4 to [^177^Lu]DOTA-r-a-ABA-CPCR4, and this would not be expected to have detectable impact on the late-phase performance of [^177^Lu]DOTA-r-a-ABA-*iodo*CPCR4.

## Summary and Conclusion

Overall, from the various compounds investigated in this study, [^177^Lu]DOTA-r-a-ABA-CPCR4 has emerged as a next lead candidate for pentapeptide-based, second-generation CXCR4-targeted therapeutic ligands. Its *in vitro* and *in vivo* CXCR4 binding characteristics and promising tumor uptake, alongside with an optimized general pharmacokinetic profile additively reflect the separate optimization steps that were implemented based on the structure-activity relationships established in this study:

- the ABA-for-AMBA-substitution in the linker (as compared to PentixaFor/PentixaTher), leading to enhanced total cellular uptake and tracer internalization;
- the introduction of a two-amino-acid extension into the linker unit, providing substantially higher flexibility towards structural variations at the far end of the linker, as exemplified by the tolerance of various M^3+^-DOTA complexes without losses in CXCR4 affinity;
- the introduction of a basic amino acid into this two-amino-acid extension, leading to a 10-fold increase in hCXCR4 affinity compared to [^177^Lu]PentixaTher, i.e. more speficially;
- the use of the optimized r-a-ABA-linker, which obliterates the necessity for Tyr^1^-iodination of the pentapeptide core to maintain high receptor affinity (such as in [^177^Lu]PentixaTher). By this modification, the undesirable side effects of using the more lipophilic *iodo*CPCR4 peptide core on general tracer pharmacokinetics can be avoided.

As a consequence, due to its improved CXCR4 targeting *in vitro* and *in vivo*, leading to higher tumor/non-tumor ratios compared to [^177^Lu]PentixaTher, [^177^Lu]DOTA-r-a-ABA-CPCR4 may have potential as a second generation CXCR4-targeted therapeutic agent and thus will be further evaluated in preclinical dosimetry studies. Generally, the structure activity studies performed in this study, leading to the optimized r-a-ABA linker structure, have provided valuable insights into the various structural and physicochemical aspects that need to be taken into account during the optimization of CXCR4-targeted peptide probes, and these insights will be implemented in our ongoing efforts to develop CXCR4-targeted probes for a broad scope of applications in molecular imaging and therapy.

## Supplementary Material

Supplementary methods, data, figures and tables.

## Figures and Tables

**Table 1:** Binding affinities (IC_50_ in nM) of [^nat^Ga]PentixaFor, [^nat^Lu]PentixaTher and selected ^nat^Lu-complexes of analogs with modified linker structure to murine CXCR4 (mCXCR4). Affinities to mCXCR4 were determined using Eµ-myc 1080 mouse lymphoma cells (200.000 cells/sample) and [^125^I]CPCR4.3 56 as radioligand. Each experiment was performed in triplicate, and results are means ± SD from a minimum of three separate experiments.

Peptide	IC_50_ [nM] to mCXCR4
[^nat^Ga]PentixaFor (entry 1)	>1000
[^nat^Lu]PentixaTher (entry 2)	567 ± 62
[^nat^Lu]DOTA-k-G-ABA-*iodo*CPCR4 (entry 10)	61 ± 17
[^nat^Lu]DOTA-r-G-ABA-*iodo*CPCR4 (entry 12)	37 ± 3
[^nat^Lu]DOTA-r-a-ABA-CPCR4 (entry 13)	182 ± 26
[^nat^Lu]DOTA-r-a-ABA-*iodo*CPCR4 (entry 14)	49 ± 1

Entry numbers in parentheses relate to the numbering in Figure 2 and are given to facilitate comparison to hCXCR4 data.

**Table 2:** Internalization and externalization characteristics of [^177^Lu]PentixaTher and selected linker-modified analogs. Total cellular uptake of the respective radioligands (1 nM) and the percentage of internalized ligand were determined using hCXCR4 expressing Chem-1 cells (100,000 cells/well, 60 min at 37°C). Ligand release and recycling within 60 min at 37°C (following internalization for 120 min at 37°C) was determined in the same cell line under conditions allowing (medium only) and inhibiting ligand recycling (10 µM AMD3100).

Peptide	Cellular uptake		Cellular retention	
	Total uptake [% of applied activity]	Internalized [% of total uptake]	Recycling inhibited [% of cellular activity at t=0]	Recycling allowed [% of cellular activity at t=0]
[^177^Lu]PentixaTher (entry 2)	10.0 ± 1.3	47 ± 16	21.8 ± 0.8	41.0 ± 1.0
[^177^Lu]DOTA-ABA-*iodo*CPCR4 (entry 3)	17.4 ± 1.9	78 ± 11	25.1 ± 0.4	41.3 ± 0.6
[^177^Lu]DOTA-r-G-ABA-CPCR4 (entry 11)	26.4 ± 1.6	51 ± 6	21.8 ± 0.4	82.3 ± 0.8
[^177^Lu]DOTA-r-G-ABA-*iodo*CPCR4 (entry 12)	38.3 ± 2.0	90 ± 8	21.8 ± 0.8	80.5 ± 2.2
[^177^Lu]DOTA-r-a-ABA-CPCR4 (entry 13)	43.1 ± 1.1	65 ± 6	14.9 ± 1.1	93.1 ± 6.3
[^177^Lu]DOTA-r-a-ABA-*iodo*CPCR4 (entry 14)	40.4 ± 1.5	91 ± 4	27.6 ± 1.0	85.8 ± 1.0

All data are corrected for non-specific binding/internalization in the presence of 100 μM AMD3100 and are expressed as mean ± SD (n = 3).

**Table 3:** Lipophilicity (log P_O/W_) of the reference ligands [^68^Ga]PentixaFor and [^177^Lu]PentixaTher and of selected ^68^Ga/^177^Lu-labeled ligands with modified linker structure. Data are means ± SD (n=6).

Peptide	log P_O/PBS_
[^68^Ga]PentixaFor	-2.9 ± 0.08
[^177^Lu]PentixaTher	-1.8 ± 0.20
[^68^Ga]DOTA-r-G-ABA-*iodo*CPCR4	-3.0 ± 0.02
[^177^Lu]DOTA-r-G-ABA-*iodo*CPCR4	-2.7 ± 0.05
[^68^Ga]DOTA-r-a-ABA-CPCR4	-3.6 ± 0.06
[^177^Lu]DOTA-r-a-ABA-CPCR4	-3.0 ± 0.13
[^68^Ga]DOTA-r-a-ABA-*iodo*CPCR4	-3.3 ± 0.02
[^177^Lu]DOTA-r-a-ABA-*iodo*CPCR4	-2.8 ± 0.04

**Table 4:** Tumor/organ ratios for [^177^Lu]PentixaTher, [^177^Lu]DOTA-r-a-ABA-CPCR4 and [^177^Lu]DOTA-r-a-ABA-iodoCPCR4 in Daudi xenograft bearing CB-17 SCID mice at **48h p.i.** (groups of n=4-5 animals). Data are means ± SD.

	[^177^Lu]PentixaTher	[^177^Lu]DOTA-r-a-ABA-CPCR4	[^177^Lu]DOTA-r-a-ABA-*iodo*CPCR4
t/blood	201 ± 27	949 ± 146	320 ± 104
t/heart	31 ± 4	111 ± 20	42 ± 7
t/lung	16 ± 5	47 ± 22	14 ± 2
t/liver	0.4 ± 0.1	1.0 ± 0.2	0.6 ± 0.1
t/spleen	2.1 ± 0.5	8.3 ± 2.8	3.0 ± 0.7
t/stomach	27 ± 6	46 ± 32	58 ± 12
t/small intestine	29 ± 6	55 ± 36	50 ± 20
t/colon	13 ± 5	20 ± 10	19 ± 13
t/kidney	2.7 ± 0.4	2.9 ± 0.4	3.1 ± 0.9
t/muscle	85 ± 15	413 ± 100	226 ± 36
