# Supplementary material for: A new class of PentixaFor- and PentixaTher-based theranostic agents with enhanced CXCR4-targeting efficiency
Source: Theranostics. 2020 Jul 9;10(18):8264–80. doi: 10.7150/thno.45537 (PMC7381729; doi:10.7150/thno.45537)
Supplement: Supplementary file 1 — Supplementary methods, data, figures and tables. [file thnov10p8264s1.pdf]

Supplementary Material

Peptide Synthesis

General information

Fmoc-(9-fluorenylmethoxycarbonyl-) and all other protected amino acid analogues were purchased from Iris Biotech (Marktredwitz, Germany) or Bachem (Bubendorf, Switzerland). Tritylchloride polystyrene (TCP) resin was obtained from PepChem (Tübingen, Germany). The chelators DOTA and DOTAGA-anhydride were purchased from Chematech (Dijon, France). Solvents and all other organic reagents were obtained from Sigma-Aldrich (Munich, Germany) or VWR (Radnor, USA). Solid phase peptide synthesis was carried out manually using an Intelli-Mixer syringe shaker (Neolab, Heidelberg, Germany). Analytical reversed-phase high performance liquid chromatography (RP-HPLC) was performed on a Nucleosil 100 C18 (5 µm, 125 × 4.0 mm) column (CS GmbH, Langerwehe, Germany) using a *Shimadzu Corp.* (Kyoto, Japan) system with two LC-20AP gradient pumps, a CBM-20A communication module, an CTO-20A column oven and a SPD-20A UV/VIS detector. For semi-preparative HPLC, a similar system from *Shimadzu Corp.* (Kyoto, Japan) with a Multospher 100 RP 18 column (5 µm, 250 *x* 10 mm, *CS Chromatographie GmbH*, Langerwehe, Germany) with 5 mL/min flow rate was utilized. For data analysis, the Lab Solutions software by *Shimadzu Corp.* was used. The peptides were eluted applying different gradients of 0.1% (v/v) trifluoroacetic acid (TFA) in H_2O (solvent A) and 0.1% TFA (v/v) in acetonitrile (solvent B) at a constant flow of 1 mL/min or 5 mL/min for purification (specific gradients are cited in the text). Both retention times t_R as well as the capacity factors K' are cited in the text. Electrospray ionization mass spectrometry (ESI-MS) was performed with a Varian 500-MS IT mass spectrometer (Agilent Technologies, Santa Clara, USA).

Fmoc solid-phase peptide synthesis

For the synthesis of the cyclic pentapeptide core cyclo(D-Tyr^1-N(Me)-D-Orn^2-Arg^3-2-Nal^4-Gly^5) (**CPCR4**) a previously reported Fmoc based solid-phase peptide synthesis (SPPS) protocol was used [1, 2].
HPLC (15 - 55% B in 15 min): t_R = 7.8 min; K´ = 4.2; calculated monoistotopic mass ($C_{36}H_{47}N_9O_6$): 701.36; found (ESI-MS): m/z = 702.7 $[M+H]^+$, 351.8 $[M+2H]^{2+}$.

Solution phase derivatization with AMBA and ABA

Deprotected CPCR4 was reacted with either 4-(Fmoc-aminomethyl)benzoic acid (Fmoc-AMBA) or 4-(Boc-amino)benzoic acid (Boc-ABA) using HOAt (1.5 eg), DIC (1.5 eq) and DIPEA (4.5 eq.) in DMF as coupling reagents. Upon subsequent deprotection of the amino function, the crude peptide was either directly

conjugated with DOTA or DOTAGA (see below) or further reacted with an additional Fmoc-amino acid (Fmoc-D-Asp, Fmoc-Gly) using the same coupling conditions, followed by Fmoc-deprotection and subsequent DOTA/DOTAGA-functionalization. All three-component linkers (ABA-X-Y-) were assembled using solid phase peptide synthesis (see below).

Synthesis of the linear linker units

The linear peptides were assembled on the solid support using a standard Fmoc-based peptide synthesis protocol. Briefly, TCP-resin was loaded with 1.8 eq of Fmoc-4-aminobenzoic acid (Fmoc-ABA). To this end, Fmoc-ABA was dissolved in dry DCM, 1.5 eq of DIPEA were added, and the suspension was added to the dry resin. After 15 min, another 2.5 eq of DIPEA were added and stirring was continued for 90 min at RT. Then, 1.5 mL methanol (MeOH) per gram resin was added to cap unreacted tritylchloride groups. After 15 min, the resin was filtered, washed twice with DCM, DMF and MeOH, respectively, and dried in vacuum. Loading of resin-bound Fmoc-ABA was calculated from the weight difference. Assembly of the peptide sequences was performed according the standard Fmoc-protocol used for the synthesis of CPCR4. Cleavage of the Fmoc-protected linkers from the resin (2 × 30 min) and concomitant ^{t}Bu/Pbf-deprotection was performed using a mixture (v/v/v) of 95% TFA, 2.5% triisobutylsilane (TIBS) and 2.5% water. The combined product solutions were then concentrated *in vacuo*, the crude peptides were precipitated using diethyl ether and were then dried *in vacuo* before HPLC purification.

Conjugation of ABA-based linkers to CPCR4

The linear ABA-based, Fmoc-protected peptide linkers (1.5 eq) were added to a solution of TBTU (1.5 eq), HOBt (1.5 eq) and DIPEA (4.5 eq) in DMF, were allowed to preactivate for 15 min and were then added to unprotected CPCR4 (1 eq.) in DMF. After 2h, the solvent was evaporated under reduced pressure and the Fmoc-peptide was treated with 20 % piperidin in DMF (v/v) for 30 min. After deprotection, the solvent was evaporated under reduced pressure and the peptide was purified using semi-preparative HPLC.

Functionalization with DOTA and DOTAGA

Final DOTA conjugation was performed according to a method published previously [3]. For DOTAGA conjugation, DOTAGA-anhydride (1.5 eq) and DIPEA (10 eq) in DMF were added to the respective N-terminally deprotected CPCR4-Linker constructs in DMF. After completion of the reaction, the solvent was evaporated under reduced pressure, and the concentrated product solution was directly used for semi-preparative HPLC purification.

Solution phase iodination of D-Tyr[1] in CPCR4-based ligands

The purified chelator-conjugated ligands were iodinated employing an optimized, mild direct iodination method recently developed in our group [4].

Preparation of the ^{nat}Ga-, ^{nat}Lu-, ^{nat}Y and ^{nat}Bi-complexes

<u>^{nat}Ga-complexes:</u> For the preparation of the ^{nat}Ga complexes, equal volumes of a 2 mM solution of $Ga(NO_3)_3$ in water and a 2 mM solution of the respective DOTA- or DOTAGA peptide in water were mixed and heated to 90°C for 30 min. After cooling, the $^{nat}Ga^{III}$-chelate formation was confirmed using RP-HPLC and MS. The resulting 1 mM solutions of the respective ^{nat}Ga-complexes were used as such for the preparation of dilution series for binding studies.

<u>^{nat}Lu-, ^{nat}Y- and ^{nat}Bi-complexes:</u> The ^{nat}Lu-, ^{nat}Y- and ^{nat}Bi-complexes of the DOTA- and DOTAGA-conjugates in this study were prepared by dissolving 200-500 µg of peptide either in 20 mM $LuCl_3$ or YCl_3 in 0.01 M HCl or in 5mM $Bi(OAc)_3$ in 0.5 M NaOAc (pH = 4.5), respectively, to yield a final peptide concentration of 1 mM. After heating to 95°C for 30 min in a sealed tube, RP-HPLC analysis revealed quantitative complex formation. The product solutions were used as such for the preparation of dilution series for binding studies.

Analytical Data for the metal-free DOTA- and DOTAGA-compounds

<u>cyclo[D-Tyr-N(Me)-D-Orn(AMBA-DOTA)-Arg-2-Nal-Gly], **DOTA-AMBA-CPCR4, PentixaFor (1)**:</u>

HPLC (15-45% B in 15 min): t_R = 9.0 min; κ' = 5.4. Calculated monoisotopic mass ($C_{60}H_{80}N_{14}O_{14}$): 1220.60; found: *m/z* = 1221.9 $[M+H]^+$, 611.2 $[M+2H]^{2+}$.

<u>cyclo[(3-iodo)D-Tyr-N(Me)-D-Orn(AMBA-DOTA)-Arg-2-Nal-Gly], **DOTA-AMBA-iodoCPCR4, PentixaTher (2)**:</u>

HPLC (15-45% B in 15 min): t_R = 10.6 min; κ' = 5.2. Calculated monoisotopic mass ($C_{60}H_{79}IN_{14}O_{14}$): 1346.49; found: *m/z* = 1347.7 $[M+H]^+$, 737.5 $[M+2H]^{2+}$, 1369.7 $[M+Na]^+$.

<u>cyclo[(3-iodo)D-Tyr-N(Me)-D-Orn(ABA-DOTA)-Arg-2-Nal-Gly], **DOTA-ABA-iodoCPCR4 (3)**:</u>

HPLC (35-60% B in 15 min): t_R = 6.8 min; κ' = 2.7. Calculated monoisotopic mass ($C_{59}H_{77}IN_{14}O_{14}$): 1332.48; found: *m/z* = 1333.9 $[M+H]^+$, 667.8 $[M+2H]^{2+}$, 1355.8 $[M+Na]^+$.

<u>cyclo[(3-iodo)D-Tyr-N(Me)-D-Orn(ABA-DOTAGA)-Arg-2-Nal-Gly], **DOTAGA-ABA-iodoCPCR4 (4)**:</u>

HPLC (35-60% B in 15 min): t_R = 9.2 min; κ' = 4.1. Calculated monoisotopic mass ($C_{62}H_{81}IN_{14}O_{16}$): 1404.50; found: *m/z* = 1405.9 $[M+H]^+$, 703.7 $[M+2H]^{2+}$.

cyclo[(3-iodo)D-Tyr-N(Me)-D-Orn(ABA-D-Asp-DOTA)-Arg-2-Nal-Gly], **DOTA-d-ABA-iodoCPCR4 (5)**:

HPLC (25-55% B in 15 min): t_R = 8.3 min; κ' = 4.9. Calculated monoisotopic mass ($C_{63}H_{82}IN_{15}O_{17}$): 1447.51; found: *m/z* = 1448.8 $[M+H]^+$, 725.3 $[M+2H]^{2+}$.

cyclo[(3-iodo)D-Tyr-N(Me)-D-Orn(ABA-Gly-DOTA)-Arg-2-Nal-Gly], **DOTA-G-ABA-iodoCPCR4 (6)**:

HPLC (35-60% B in 15 min): t_R = 8.8 min; κ' = 3.8. Calculated monoisotopic mass ($C_{61}H_{80}IN_{15}O_{15}$): 1389.50; found: *m/z* = 1390.9 $[M+H]^+$, 696.3 $[M+2H]^{2+}$.

cyclo[(3-iodo)D-Tyr-N(Me)-D-Orn(ABA-Gly-DOTAGA)-Arg-2-Nal-Gly], **DOTAGA-G-ABA-iodoCPCR4 (7)**:

HPLC (30-55% B in 15 min): t_R = 12.2 min; κ' = 5.7. Calculated monoisotopic mass ($C_{64}H_{84}IN_{15}O_{17}$): 1461.52; found: *m/z* = 1462.8 $[M+H]^+$, 732.2 $[M+2H]^{2+}$.

cyclo[(3-iodo)D-Tyr-N(Me)-D-Orn(ABA-Gly-D-Dap-DOTA)-Arg-2-Nal-Gly], **DOTA-dap-G-ABA-iodo-CPCR4 (8)**:

HPLC (25-65% B in 15 min): t_R = 11.89 min; κ' = 5.2. Calculated monoisotopic mass ($C_{64}H_{86}IN_{17}O_{16}$): 1475.55; found: *m/z* = 1476.8 $[M+H]^+$, 739.3 $[M+2H]^{2+}$.

cyclo[D-Tyr-N(Me)-D-Orn(ABA-Gly-D-Lys-DOTA)-Arg-2-Nal-Gly], **DOTA-k-G-ABA-CPCR4 (9)**:

HPLC (15-55% B in 15 min): t_R = 7.9 min; κ' = 7.0. Calculated monoisotopic mass ($C_{67}H_{93}N_{17}O_{16}$): 1391.70; found: *m/z* = 1393.2 $[M+H]^+$.

cyclo[(3-iodo)D-Tyr-N(Me)-D-Orn(ABA-Gly-D-Lys-DOTA)-Arg-2-Nal-Gly], **DOTA-k-G-ABA-iodoCPCR4 (10)**:

HPLC (25-55% B in 15 min): t_R = 12.8 min; κ' = 7.0. Calculated monoisotopic mass ($C_{67}H_{92}IN_{17}O_{16}$): 1517.60; found: *m/z* = 1519.1 $[M+H]^+$, 760.4 $[M+2H]^{2+}$.

cyclo[D-Tyr-N(Me)-D-Orn(ABA-Gly-D-Arg-DOTA)-Arg-2-Nal-Gly], **DOTA-r-G-ABA-CPCR4 (11)**:

HPLC (15-55% B in 15 min): t_R = 8.2 min; κ' = 4.8. Calculated monoisotopic mass ($C_{67}H_{93}N_{19}O_{16}$): 1419.70; found: *m/z* = 1421.5 $[M+H]^+$.

cyclo[(3-iodo)D-Tyr-N(Me)-D-Orn(ABA-Gly-D-Arg-DOTA)-Arg-2-Nal-Gly], **DOTA-r-G-ABA-iodoCPCR4 (12)**:

HPLC (25-55% B in 15 min): t_R = 13.3 min; κ' = 5.6. Calculated monoisotopic mass ($C_{67}H_{92}IN_{19}O_{16}$): 1545.60; found: *m/z* = 1547.2 $[M+H]^+$, 774.5 $[M+2H]^{2+}$.

cyclo[D-Tyr-N(Me)-D-Orn(DOTA-D-Arg-D-Ala-ABA)-Arg-2-Nal-Gly], **DOTA-r-a-ABA-CPCR4** (**13**):

HPLC (15-55% B in 15 min): t_R = 8.0 min; κ' = 3.0. Calculated monoisotopic mass ($C_{68}H_{95}N_{19}O_{16}$): 1433.72; found: *m/z* = 1435.1 $[M+H]^+$, 717.3 $[M+2H]^{2+}$.

cyclo[(3-iodo)D-Tyr-N(Me)-D-Orn(DOTA-D-Arg-D-Ala-ABA)-Arg-2Nal-Gly], **DOTA-r-a-ABA-iodo-CPCR4** (**14**):

HPLC (25-55% B in 15 min): t_R = 12.1 min; κ' = 7.0. Calculated monoisotopic mass ($C_{68}H_{94}IN_{19}O_{16}$): 1559.62; found: *m/z* = 1560.2 $[M+H]^+$, 781.5 $[M+2H]^{2+}$.

Analytical Data for the ^{nat}Ga-compounds

[^{nat}Ga]DOTA-AMBA-CPCR4, [^{nat}Ga]PentixaFor (**1**):

HPLC (30-55% B in 15 min): t_R = 9.0 min; κ'= 5.0; Calculated monoisotopic mass ($C_{60}H_{78}GaN_{14}O_{14}$): 1,287.50; found by ESI-MS: *m/z* = 1,287.7 $[M+H]^+$, 1,311.7 $[M+Na]^+$.

[^{nat}Ga]DOTA-AMBA-iodoCPCR4, [^{nat}Ga]PentixaTher (**2**):

HPLC (30-55% B in 15 min): t_R = 10.8 min; κ'= 5.8; Calculated monoisotopic mass ($C_{60}H_{77}GaIN_{14}O_{14}$): 1,413.40; found by ESI-MS: *m/z* = 1,414.9 $[M+H]^+$, 1,437.2 $[M+Na]^+$, 707.6 $[M+2H]^{2+}$.

[^{nat}Ga]DOTA-ABA-iodoCPCR4 (**3**):

HPLC (35-60% B in 15 min): t_R = 8.1 min; κ'= 4.4; Calculated monoisotopic mass ($C_{59}H_{74}ILuN_{14}O_{14}$): 1,504.40; found by ESI-MS: *m/z* = 1,505.9 $[M+H]^+$, 1,527.7 $[M+Na]^+$, 1,543.7 $[M+K]^+$, 753.6 $[M+2H]^{2+}$.

[^{nat}Ga]DOTAGA-ABA-iodoCPCR4 (**4**):

HPLC (35-60% B in 15 min): t_R = 7.0 min; κ'= 4.0; Calculated monoisotopic mass ($C_{62}H_{79}IGaN_{14}O_{16}$): 1471.42, found by ESI-MS: *m/z* = 1472.6 $[M+H]^+$, 736.8 $[M+2H]^{2+}$.

[^{nat}Ga]DOTA-G-ABA-iodoCPCR4 (**6**):

HPLC (35-60% B in 15 min): t_R = 9.1 min; κ'= 5.5; Calculated monoisotopic mass ($C_{61}H_{78}GaIN_{15}O_{15}$): 1456.41, found by ESI-MS: *m/z* = 1456.8 $[M+H]^+$, 1,478.5 $[M+Na]^+$.

[^{nat}Ga]DOTAGA-G-ABA-iodoCPCR4 (**7**):

HPLC (30-55% B in 15 min): t_R = 12.8 min; κ'= 6.1; Calculated monoisotopic mass ($C_{64}H_{82}GaIN_{15}O_{17}$): 1528.43, found by ESI-MS: *m/z* = 1528.4 $[M+H]^+$, 1552.7 $[M+Na]^+$, 765.9 $[M+2H]^{2+}$, 776.7 $[M+H+Na]^{2+}$.

[^{nat}Ga]DOTA-dap-G-ABA-iodo-CPCR4 (**8**):

HPLC (25-65% B in 15 min): t_R = 11.4 min; κ'= 5.3; Calculated monoisotopic mass ($C_{64}H_{84}GaIN_{17}O_{16}$): 1542.46, found by ESI-MS: *m/z* = 1544.8 $[M+H]^+$, 772.4 $[M+2H]^{2+}$.

[^{nat}Ga]DOTA-k-G-ABA-CPCR4 (**9**):

HPLC (15-55% B in 15 min): t_R = 7.6 min; κ'= 3.0; Calculated monoisotopic mass ($C_{67}H_{91}GaN_{17}O_{16}$): 1458.61, found by ESI-MS: *m/z* = 1459.8 $[M+H]^+$, 730.2 $[M+2H]^{2+}$.

[^{nat}Ga]DOTA-k-G-ABA-iodoCPCR4 (**10**):

HPLC (25-55% B in 15 min): t_R = 12.9 min; κ'= 6.1; Calculated monoisotopic mass ($C_{67}H_{90}GaIN_{17}O_{16}$): 1584.51, found by ESI-MS: *m/z* = 1585.0 $[M+H]^+$, 1608.9 $[M+Na]^+$, 793.7 $[M+2H]^{2+}$.

[^{nat}Ga]DOTA-r-G-ABA-CPCR4 (**11**):

HPLC (15-55% B in 15 min): t_R = 7.7 min; κ'= 3.2; Calculated monoisotopic mass ($C_{67}H_{91}GaN_{19}O_{16}$): 1468.61, found by ESI-MS: *m/z* = 745.3 $[M+2H]^{2+}$.

[^{nat}Ga]DOTA-r-G-ABA-iodoCPCR4 (**12**):

HPLC (25-55% B in 15 min): t_R = 12.6 min; κ'= 6.8; Calculated monoisotopic mass ($C_{67}H_{90}GaIN_{19}O_{16}$): 1612.51, found by ESI-MS: *m/z* = 1614.9 $[M+H]^+$, 808.0 $[M+2H]^{2+}$.

[^{nat}Ga]DOTA-r-a-ABA-CPCR4 (**13**):

HPLC (15-55% B in 15 min): t_R = 6.8 min; κ'=3.5; Calculated monoisotopic mass ($C_{68}H_{93}GaN_{19}O_{16}$): 1500.63, found by ESI-MS: *m/z* = 751.5 $[M+2H]^{2+}$, 787.2 $[M+2K]^{2+}$.

[^{nat}Ga]DOTA-r-a-ABA-iodo-CPCR4 (**14**):

HPLC (25%-55% B in 15 min): t_R = 7.9 min; κ' = 3.9; Calculated monoisotopic mass ($C_{68}H_{92}GaIN_{19}O_{16}$): 1626.53, found by ESI-MS: *m/z* = 1626.8 $[M+H]^+$, 815.0 $[M+2H]^{2+}$.

Analytical Data for the ^{nat}Lu-compounds

[^{nat}Lu]DOTA-AMBA-iodoCPCR4, [^{nat}Lu]PentixaTher (**2**):

HPLC (15-55% B in 15 min): t_R = 6.3 min; κ'= 4.3; Calculated monoisotopic mass ($C_{60}H_{76}ILuN_{14}O_{14}$): 1,518.41; found by ESI-MS: *m/z* = 1,520.1 $[M+H]^+$, 760.8 $[M+2H]^{2+}$.

[^{nat}Lu]DOTA-ABA-iodoCPCR4 (**3**):

HPLC (35-60% B in 15 min): t_R = 8.1 min; κ'= 4.4; Calculated monoisotopic mass ($C_{59}H_{74}ILuN_{14}O_{14}$): 1,504.40; found by ESI-MS: *m/z* = 1,505.9 $[M+H]^+$, 1,527.7 $[M+Na]^+$, 1,543.7 $[M+K]^+$, 753.6 $[M+2H]^{2+}$.

[^{nat}Lu]DOTAGA-ABA-iodoCPCR4 (**4**):

HPLC (35 to 60% B in 15 min): t_R = 9.2 min; κ'= 4.1; Calculated monoisotopic mass ($C_{62}H_{78}ILuN_{14}O_{16}$): 1576.42, found by ESI-MS: *m/z* = 1577.6 $[M+H]^+$, 1,600.5 $[M+Na]^+$, 789.5 $[M+2H]^{2+}$, 800.5 $[M+H+Na]^{2+}$.

[^{nat}Lu]DOTA-d-ABA-iodoCPCR4 (**5**):

HPLC (35-65% B in 15 min): t_R = 8.0 min; κ'= 3.4; Calculated monoisotopic mass ($C_{63}H_{79}ILuN_{15}O_{17}$): 1619.42, found by ESI-MS: *m/z* = 1620.9 $[M+H]^+$, 1,642.8 $[M+Na]^+$, 811.2 $[M+2H]^{2+}$, 822.0 $[M+H+Na]^{2+}$.

[^{nat}Lu]DOTA-G-ABA-iodoCPCR4 (**6**):

HPLC (35-60% B in 15 min): t_R = 9.1 min; κ'= 5.5; Calculated monoisotopic mass ($C_{61}H_{77}ILuN_{15}O_{15}$): 1561.42, found by ESI-MS: *m/z* = 1562.6 $[M+H]^+$, 1,584.5 $[M+Na]^+$, 782.0 $[M+2H]^{2+}$, 792.9 $[M+H+Na]^{2+}$.

[^{nat}Lu]DOTAGA-G-ABA-iodoCPCR4 (**7**):

HPLC (35-60% B in 15 min): t_R = 9.3 min; κ'= 4.2; Calculated monoisotopic mass ($C_{64}H_{81}ILuN_{15}O_{17}$): 1633.44, found by ESI-MS: *m/z* = 1634.7 $[M+H]^+$, 1,656.6 $[M+Na]^+$, 818.1 $[M+2H]^{2+}$, 829.0 $[M+H+Na]^{2+}$.

[^{nat}Lu]DOTA-dap-G-ABA-iodo-CPCR4 (**8**):

HPLC (25-65% B in 15 min): t_R = 10.8 min; κ'= 9.8; Calculated monoisotopic mass ($C_{64}H_{83}ILuN_{17}O_{16}$): 1647.47, found by ESI-MS: *m/z* = 1648.9 $[M+H]^+$, 825.2 $[M+2H]^{2+}$, 860.5 $[M+2K]^{2+}$.

[^{nat}Lu]DOTA-k-G-ABA-CPCR4 (**9**):

HPLC (15-55% B in 15 min): t_R = 7.5 min; κ'= 4.0; Calculated monoisotopic mass ($C_{67}H_{90}LuN_{17}O_{16}$): 1563.62, found by ESI-MS: *m/z* = 1565.4 $[M+H]^+$.

[^{nat}Lu]DOTA-k-G-ABA-iodoCPCR4 (**10**):

HPLC (25-55% B in 15 min): t_R = 13.1 min; κ'= 7.7; Calculated monoisotopic mass ($C_{67}H_{89}ILuN_{17}O_{16}$): 1689.51, found by ESI-MS: *m/z* = 1691.8 $[M+H]^+$, 1712.7 $[M+Na]^+$, 846.4 $[M+2H]^{2+}$, 857.1 $[M+H+Na]^{2+}$.

[^{nat}Lu]DOTA-r-G-ABA-CPCR4 (**11**):

HPLC (15-55% B in 15 min): t_R = 7.2 min; κ'= 4.1; Calculated monoisotopic mass ($C_{67}H_{90}LuN_{19}O_{16}$): 1591.62, found by ESI-MS: *m/z* = 1592.5 $[M+H]^+$, 1631.0 $[M+K]^+$.

[^{nat}Lu]DOTA-r-G-ABA-iodoCPCR4 (**12**):

HPLC (25-55% B in 15 min): t_R = 13.4 min; κ'= 7.9; Calculated monoisotopic mass ($C_{67}H_{89}IN_{19}O_{16}$): 1717.52, found by ESI-MS: *m/z* = 1719.9 $[M+H]^+$, 860.5 $[M+2H]^{2+}$, 871.1 $[M+H+Na]^{2+}$, 895.7 $[M+2K]^{2+}$.

[^{nat}Lu]DOTA-r-a-ABA-CPCR4 (**13**):

HPLC (15-55% B in 15 min): t_R = 7.5 min; κ'= 4.0; Calculated monoisotopic mass ($C_{68}H_{92}LuN_{19}O_{16}$): 1605.64, found by ESI-MS: *m/z* = 1607.2 $[M+H]^+$, 804.6 $[M+2H]^{2+}$.

[^{nat}Lu]DOTA-r-a-ABA-iodo-CPCR4 (**14**):

HPLC (25-55% B in 15 min): t_R = 6.5 min; κ'= 2.3; Calculated monoisotopic mass ($C_{68}H_{91}ILuN_{19}O_{16}$): 1731.53, found by ESI-MS: *m/z* = 1732.8 $[M+H]^+$, 867.3 $[M+2H]^{2+}$, 902.6 $[M+2K]^{2+}$.

Analytical Data for the ^{nat}Y-compounds

[^{nat}Y]DOTA-AMBA-iodoCPCR4, [^{nat}Y]PentixaTher (**2**):

HPLC (15-55% B in 15 min): t_R = 6.5 min; κ'= 3.6; Calculated monoisotopic mass ($C_{60}H_{76}IN_{14}O_{14}Y$): 1432.38; found by ESI-MS: *m/z* = 1,433.6 $[M+H]^+$, 1,455.8 $[M+Na]^+$, 717.4 $[M+2H]^{2+}$.

[^{nat}Y]DOTA-ABA-iodoCPCR4 (**3**):

HPLC (35-60% B in 15 min): t_R = 4.9 min; κ'= 2.3; Calculated monoisotopic mass ($C_{59}H_{74}IN_{14}O_{14}Y$): 1418.36; found by ESI-MS: *m/z* = 1,419.8 $[M+H]^+$, 1,442.7 $[M+Na]^+$, 710.4 $[M+2H]^{2+}$.

[^{nat}Y]DOTAGA-ABA-iodoCPCR4 (**4**):

HPLC (35-60% B in 15 min): t_R = 9.2 min; κ'= 4.4; Calculated monoisotopic mass ($C_{62}H_{78}IN_{14}O_{16}Y$): 1490.38, found by ESI-MS: *m/z* = 1491.6 $[M+H]^+$, 1513.5 $[M+Na]^+$, 746.5 $[M+2H]^{2+}$, 757.4 $[M+H+Na]^{2+}$.

[^{nat}Y]DOTA-G-ABA-iodoCPCR4 (**6**):

HPLC (35-60% B in 15 min): t_R = 8.2 min; κ'= 7.2; Calculated monoisotopic mass ($C_{61}H_{77}IN_{15}O_{15}Y$): 1475.38, found by ESI-MS: *m/z* = 1476.7 $[M+H]^+$, 1498.5 $[M+Na]^+$, 739.0 $[M+2H]^{2+}$, 750.0 $[M+H+Na]^{2+}$.

[^{nat}Y]DOTAGA-G-ABA-iodoCPCR4 (**7**):

HPLC (35-65% B in 15 min): t_R = 9.2 min; κ'= 4.1; Calculated monoisotopic mass ($C_{64}H_{81}IN_{15}O_{17}Y$): 1547.40, found by ESI-MS: *m/z* = 1548.6 $[M+H]^+$, 1570.4 $[M+Na]^+$, 775.0 $[M+2H]^{2+}$, 785.9 $[M+H+Na]^{2+}$.

[^{nat}Y]DOTA-dap-G-ABA-iodo-CPCR4 (**8**):

HPLC (25-65% B in 15 min): t_R = 11.5 min; κ'= 6.1; Calculated monoisotopic mass ($C_{64}H_{83}IN_{17}O_{16}Y$): 1561.43, found by ESI-MS: *m/z* = 1562.7 $[M+H]^+$, 782.3 $[M+2H]^{2+}$, 793.0 $[M+H+Na]^{2+}$.

[^{nat}Y]DOTA-k-G-ABA-CPCR4 (**9**):

HPLC (15-55% B in 15 min): t_R = 7.8 min; κ'= 2.7; Calculated monoisotopic mass ($C_{67}H_{90}N_{17}O_{16}Y$): 1477.58, found by ESI-MS: *m/z* = 1478.9 $[M+H]^+$, 740.4 $[M+2H]^{2+}$.

[^{nat}Y]DOTA-k-G-ABA-iodoCPCR4 (**10**):

HPLC (25-55% B in 15 min): t_R = 12.9 min; κ'= 7.6; Calculated monoisotopic mass ($C_{67}H_{89}IN_{17}O_{16}Y$): 1603.48, found by ESI-MS: *m/z* = 1605.7 $[M+H]^+$, 1626.6 $[M+Na]^+$, 803.4 $[M+2H]^{2+}$, 814.1 $[M+H+Na]^{2+}$.

[^{nat}Y]DOTA-r-G-ABA-CPCR4 (**11**):

HPLC (15-55% B in 15 min): t_R = 8.0 min; κ'= 2.8; Calculated monoisotopic mass ($C_{67}H_{90}N_{19}O_{16}Y$): 1505.59, found by ESI-MS: *m/z* = 1506.2 $[M+H]^+$, 754.3 $[M+2H]^{2+}$.

[^{nat}Y]DOTA-r-G-ABA-iodoCPCR4 (**12**):

HPLC (25-55% B in 15 min): t_R = 13.3 min; κ'= 7.9; Calculated monoisotopic mass ($C_{67}H_{89}IN_{19}O_{16}Y$): 1631.48, found by ESI-MS: *m/z* = 1632.9 $[M+H]^+$, 817.4 $[M+2H]^{2+}$, 828.1 $[M+H+Na]^{2+}$, 852.7 $[M+2K]^{2+}$.

[^{nat}Y]DOTA-r-a-ABA-CPCR4 (**13**):

HPLC (15-55% B in 15 min): t_R = 7.0 min; κ'= 3.6; Calculated monoisotopic mass ($C_{68}H_{92}N_{19}O_{16}Y$): 1519.60, found by ESI-MS: *m/z* = 761.0 $[M+2H]^{2+}$.

[^{nat}Y]DOTA-r-a-ABA-iodo-CPCR4 (**14**):

HPLC (25-55% B in 15 min): t_R = 9.6 min; κ'= 2.8; Calculated monoisotopic mass ($C_{68}H_{91}IN_{19}O_{16}Y$): 1645.50, found by ESI-MS: m/z = 1646.9 $[M+H]^+$, 824.1 $[M+2H]^{2+}$, 859.5 $[M+2K]^{2+}$.

Analytical Data for the ^{nat}Bi-compounds

[^{nat}Bi]DOTA-AMBA-iodoCPCR4, [^{nat}Bi]PentixaTher (**2**):

HPLC (15-55% B in 15 min): t_R = 9.6 min; κ'= 4.3; Calculated monoisotopic mass ($C_{60}H_{76}BiIN_{14}O_{14}$): 1552.45; found by ESI-MS: m/z = 1,554.2 $[M+H]^+$, 778.2 $[M+2H]^{2+}$.

[^{nat}Bi]DOTA-ABA-iodoCPCR4 (**3**):

HPLC (15-55% B in 15 min): t_R = 9.6 min; κ'= 5.0; Calculated monoisotopic mass ($C_{59}H_{74}BiIN_{14}O_{14}$): 1538.44; found by ESI-MS: m/z = 1,540.6 $[M+H]^+$, 770.1 $[M+2H]^{2+}$.

[^{nat}Bi]DOTA-G-ABA-iodoCPCR4 (**6**):

HPLC (15-55% B in 15 min): t_R = 9.5 min; κ'= 4.3; Calculated monoisotopic mass ($C_{61}H_{77}BiIN_{15}O_{15}$): 1595.46, found by ESI-MS: m/z = 1597.4 $[M+H]^+$, 799.5 $[M+2H]^{2+}$.

[^{nat}Bi]DOTA-dap-G-ABA-iodo-CPCR4 (**8**):

HPLC (15-55% B in 15 min): t_R = 8.9 min; κ'= 3.9; Calculated monoisotopic mass ($C_{64}H_{83}BiIN_{17}O_{16}$): 1681.51, found by ESI-MS: m/z = 1682.2 $[M+H]^+$, 839.8 $[M+2H]^{2+}$.

[^{nat}Bi]DOTA-k-G-ABA-iodoCPCR4 (**10**):

HPLC (15-55% B in 15 min): t_R = 8.9 min; κ'= 3.3; Calculated monoisotopic mass ($C_{67}H_{89}BiN_{17}O_{16}$): 1724.56, found by ESI-MS: m/z = 1726.2 $[M+H]^+$, 863.2 $[M+2H]^{2+}$.

[^{nat}Bi]DOTA-r-G-ABA-iodoCPCR4 (**12**):

HPLC (15-55% B in 15 min): t_R = 9.0 min; κ'= 3.7; Calculated monoisotopic mass ($C_{67}H_{89}BiN_{19}O_{16}$): 1751.56, found by ESI-MS: m/z = 1752.3 $[M+H]^+$.

[^{nat}Bi]DOTA-r-a-ABA-iodo-CPCR4 (**14**):

HPLC (15-55% B in 15 min): t_R = 9.1 min; κ'= 4.0; Calculated monoisotopic mass ($C_{68}H_{91}BiIN_{19}O_{16}$): 1765.57, found by ESI-MS: m/z = 1767.5 $[M+H]^+$, 885.1 $[M+2H]^{2+}$.

Radiolabeling

^{68}Ga-labeling was performed as published previously [5, 6] using a ^{68}Ge/^{68}Ga generator (iTHEMBA Labs, South Africa) and a GallElut^{+} synthesis module (SCINTOMICS GmbH, Germany). Usually, 5 nmol of precursor peptide were used, yielding the ^{68}Ga-labeled peptides in specific activities of 100 to 120 GBq/µmol.

For ^{177}Lu-labeling, the peptides were dissolved in water to yield a 1 mM solution. Of this solution, the required volume was added to $^{177}LuCl_3$ in 0.04 M HCl (ITG/ITM, Garching, Germany; activity concentration: 370 MBq/500 µl) to achieve a peptide-to-^{177}Lu-activity ratio of 1 nmol peptide per 225 MBq $^{177}LuCl_3$. To this mixture, 1 M NH_4OAc was added (calculated to be 10% of total reaction volume), and the mixture was heated to 90°C for 40 min. After cooling and determination of the radiochemical purity (usually >98%), the reaction mixture was diluted with PBS to the desired activity concentration and used as such for *in vitro* and *in vivo* studies.

cAMP assay (hCXCR4)

Human glioma U87 cells overexpressing CD4 and CXCR4 (U87CD4CXCR4) cells were kept in cultivation at least one week before starting the experiment. The cells were washed and harvested according to the standard procedure for adherent cells. App. 1500 – 2000 cells per well were seeded in a 384-well plate in 2% FCS 1% penicillin 24 h before the test. *Perkin-Elmer's* LANCE cAMP assay kit (catalog #AD0262) based on time-resolved fluorescence resonance energy transfer (TR-FRET) was utilized to determine the ability of [^{nat}Lu]DOTA-r-a-ABA-*iodo*CPCR4 and [^{nat}Lu]PentixaTher vs the reference CXCL12 to inhibit cAMP production induced by 5 μM Forskolin. To comparatively investigate the (agonistic) potency of [^{nat}Lu]DOTA-r-a-ABA-*iodo*CPCR4 and [^{nat}Lu]PentixaTher for G_i production, subsequent inhibition of adenylyl cyclase and consequently cAMP production, CXCL12 was replaced by different concentrations of the test compounds. The influence of the respective compounds on intracellular cAMP concentrations were measured in a Perkin-Elmer Envision 2102 multilabel reader with the following parameters: flash energy area = low, flash energy level = 239, counting cycle = 1 ms, and ex/em = 340 nm/665 nm.

In vivo metabolite analysis

[^{177}Lu]DOTA-r-a-ABS-CPCR4 (app. 35 MBq) was injected into the tail vein of a SCID mouse under isoflurane anesthesia. The animal was sacrificed after 30 min, and samples form urine, blood, liver and kidney were taken immediately. The collected urine was diluted with PBS and directly analyzed using radio-HPLC. Parts of the liver and one kidney were snap-frozen in liquid nitrogen, homogenized using a ball mill and taken up in 0.2-1 mL PBS containing 50 µg of AMD3100. The suspensions were centrifuged

(15,000 g), and the supernatants were subjected to ultrafiltration. The respective ultrafiltrates were then analyzed via radio-HPLC (Chromolith Performance 18 E 4.6 x 100 mm; flow rate = 3 mL/min; applied gradient: 3 to 95% B in 6 min, 220 nm). The blood samples were centrifuged to separate the plasma from the blood cells. Plasma proteins were removed by precipitation with cold acetonitrile (50% (v/v), 10 min, 4 °C) and subsequent centrifugation and ultrafiltration as described before. The blood extract was also analyzed by radio-HPLC. For all extracts the extraction efficiency was determined in a ɣ-counter.

Supplementary Tables

Table S1 *Biodistribution of [^{68}Ga]DOTA-r-a-ABA-CPCR4 and [^{68}Ga]DOTA-r-a-ABA-iodoCPCR4 in Daudi (human B cell lymphoma) xenograft bearing CB-17 SCID mice at 1h p.i. (groups of n=4 animals). CXCR4-specificity of tracer uptake was determined by coinjection of 50 μg AMD3100/animal (n=3). Data are given in %iD/g and are means ± SD.*

organ	[^{68}Ga]DOTA-r-a-ABA-CPCR4 (tracer only)	[^{68}Ga]DOTA-r-a-ABA-CPCR4 (competition with AMD3100)	[^{68}Ga]DOTA-r-a-ABA-*iodo*CPCR4
**blood**	3.95 ± 0.89	2.98 ± 0.57*	3.71 ± 0.28
**heart**	1.51 ± 0.25	1.37 ± 0.33	1.56 ± 0.20
**lung**	3.15 ± 0.50	3.12 ± 0.57	2.91 ± 0.28
**liver**	5.63 ± 0.51	4.62 ± 0.56**	5.66 ± 0.40
**pancreas**	0.68 ± 0.01	0.69 ± 0.07	0.88 ± 0.06
**spleen**	2.01 ± 0.19	2.38 ± 0.23	3.02 ± 0.46
**stomach**	1.20 ± 0.10	0.97 ± 0.32	0.93 ± 0.15
**small intestine**	0.94 ± 0.13	0.94 ± 0.31	1.18 ± 0.03
**colon**	0.67 ± 0.13	0.47 ± 0.09	0.65 ± 0.29
**adrenals**	1.90 ± 1.14	1.02 ± 0.27	1.51 ± 0.18
**kidney**	11.50 ± 3.58	7.16 ± 1.62	6.25 ± 0.52
**muscle**	0.45 ± 0.05	0.40 ± 0.11	0.49 ± 0.08
**femur**	1.27 ± 0.32	0.74 ± 0.11**	1.41 ± 0.16
**tumor**	11.69 ± 1.31	2.52 ± 0.36***	8.32 ± 1.30

*Reduction of tracer uptake compared to control: * p = 0.07; ** 0.01 < p < 0.05; *** p ≤ 0.005*

Table S2 *Biodistribution of [^{177}Lu]PentixaTher, [^{177}Lu]DOTA-r-a-ABA-CPCR4 and [^{177}Lu]DOTA-r-a-ABA-iodoCPCR4 in Daudi (human B cell lymphoma) xenograft bearing CB-17 SCID mice at* ***1h p.i.*** *(groups of n=4-5 animals). CXCR4-specificity of tracer uptake was exemplarily confirmed for [^{177}Lu]DOTA-r-a-ABA-CPCR4 by coinjection of 50 μg AMD3100/animal (n=1). Data are given in %iD/g and are means ± SD.*

organ	[^{177}Lu]PentixaTher	[^{177}Lu]DOTA-r-a-ABA-CPCR4	[^{177}Lu]DOTA-r-a-ABA-CPCR4 + AMD3100	[^{177}Lu]DOTA-r-a-ABA-*iodo*CPCR4
**blood**	1.3 ± 0.4	1.50 ± 0.10	1.90	2.90 ± 0.32
**heart**	0.9 ± 0.3	0.90 ± 0.07	1.07	1.87 ± 0.32
**lung**	2.5 ± 0.7	3.53 ± 1.20	3.34	7.06 ± 0.67
**liver**	10.3 ± 0.8	11.9 ± 1.57	5.46	27.1 ± 1.87
**pancreas**	0.4 ± 0.1	0.44 ± 0.05	0.61	0.69 ± 0.14
**spleen**	1.8 ± 0.3	2.41 ± 0.34	1.11	6.72 ± 0.70
**stomach**	1.2 ± 0.3	1.72 ± 0.16	2.46	1.95 ± 0.17
**small intestine**	0.9 ± 0.2	1.23 ± 0.18	1.36	1.44 ± 0.22
**colon**	1.3 ± 1.2	0.78 ± 0.28	0.74	1.19 ± 0.35
**adrenals**	1.5 ± 0.6	1.16 ± 0.18	1.22	4.54 ± 1.06
**kidney**	3.4 ± 0.6	9.74 ± 0.91	10.3	10.3 ± 0.26
**muscle**	0.3 ± 0.1	0.28 ± 0.02	0.38	0.58 ± 0.07
**femur**	0.6 ± 0.2	0.86 ± 0.04	0.74	1.92 ± 0.20
**brain**	0.05 ± 0.01	-	-	0.08 ± 0.01
**tumor**	12.4 ± 3.7	18.3 ± 3.66	3.04	17.2 ± 2.01

Table S3 *Biodistribution of [^{177}Lu]PentixaTher, [^{177}Lu]DOTA-r-a-ABA-CPCR4 and [^{177}Lu]DOTA-r-a-ABA-iodoCPCR4 in Daudi (human B cell lymphoma) xenograft bearing CB-17 SCID mice at* ***6h p.i.*** *(groups of n=4-5 animals). Data are given in %iD/g and are means ± SD.*

organ	[^{177}Lu]PentixaTher	[^{177}Lu]DOTA-r-a-ABA-CPCR4	[^{177}Lu]DOTA-r-a-ABA-*iodo*CPCR4
**blood**	0.09 ± 0.01	0.05 ± 0.01	0.17 ± 0.09
**heart**	0.18 ± 0.02	0.13 ± 0.02	0.28 ± 0.05
**lung**	0.42 ± 0.06	0.27 ± 0.06	1.07 ± 0.26
**liver**	9.59 ± 0.69	11.3 ± 2.83	17.1 ± 3.09
**pancreas**	0.09 ± 0.01	0.08 ± 0.02	0.11 ± 0.02
**spleen**	1.35 ± 0.41	1.41 ± 0.24	2.95 ± 0.75
**stomach**	0.21 ± 0.01	0.25 ± 0.07	0.25 ± 0.09
**small intestine**	0.22 ± 0.04	0.30 ± 0.06	0.24 ± 0.06
**colon**	0.50 ± 0.16	0.65 ± 0.30	0.49 ± 0.20
**adrenals**	0.64 ± 0.13	0.51 ± 0.12	0.68 ± 0.21
**kidney**	2.52 ± 0.33	7.10 ± 2.06	3.20 ± 0.45
**muscle**	0.06 ± 0.01	0.04 ± 0.01	0.06 ± 0.01
**femur**	0.23 ± 0.02	0.30 ± 0.05	0.56 ± 0.23
**brain**	0.02 ± 0.005	-	-
**tumor**	6.79 ± 0.68	13.6 ± 3.33	12.5 ± 2.56

Table S4 *Biodistribution of [^{177}Lu]PentixaTher, [^{177}Lu]DOTA-r-a-ABA-CPCR4 and [^{177}Lu]DOTA-r-a-ABA-iodoCPCR4 in Daudi (human B cell lymphoma) xenograft bearing CB-17 SCID mice at* ***48h p.i.*** *(groups of n=4-5 animals). Data are given in %iD/g and are means ± SD.*

organ	[^{177}Lu]PentixaTher	[^{177}Lu]DOTA-r-a -ABA-CPCR4	[^{177}Lu]DOTA-r-a-ABA-*iodo*CPCR4
**blood**	0.02 ± 0.001	0.01 ± 0.00	0.03 ± 0.01
**heart**	0.11 ± 0.01	0.08 ± 0.01	0.19 ± 0.02
**lung**	0.21 ± 0.06	0.19 ± 0.08	0.57 ± 0.04
**liver**	8.25 ± 2.23	8.99 ± 1.62	14.6 ± 2.00
**pancreas**	0.08 ± 0.01	0.07 ± 0.01	0.09 ± 0.01
**spleen**	1.58 ± 0.33	1.06 ± 0.33	2.72 ± 0.50
**stomach**	0.12 ± 0.02	0.19 ± 0.13	0.14 ± 0.02
**small intestine**	0.11 ± 0.02	0.16 ± 0.11	0.16 ± 0.06
**colon**	0.25 ± 0.10	0.44 ± 0.22	0.43 ± 0.30
**adrenals**	0.56 ± 0.08	0.33 ± 0.08	0.60 ± 0.12
**kidney**	1.20 ± 0.13	3.05 ± 0.15	2.58 ± 0.69
**muscle**	0.04 ± 0.005	0.02 ± 0.00	0.04 ± 0.00
**femur**	0.22 ± 0.05	0.29 ± 0.05	0.34 ± 0.08
**brain**	0.007 ± 0.002	-	-
**tumor**	3.27 ± 0.41	8.81 ± 0.99	8.11 ± 0.96

Supplementary Figures

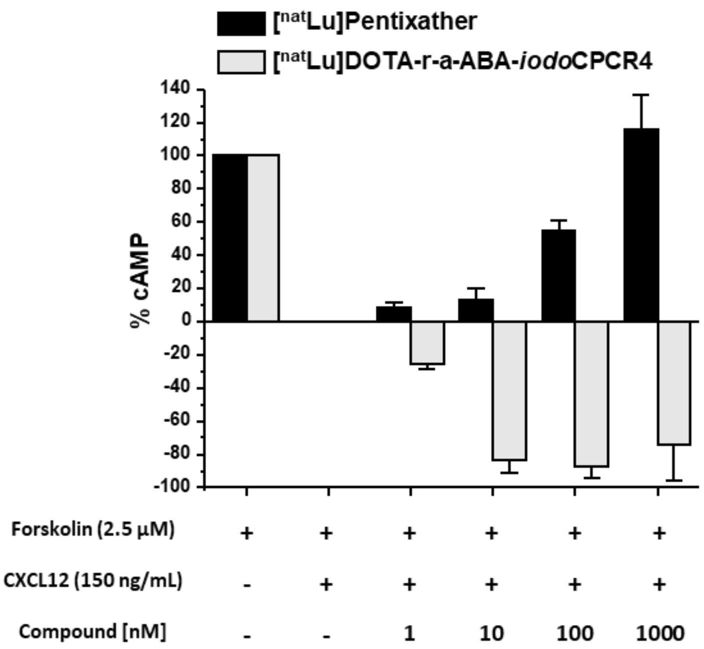

Figure S1 *Functional cAMP assay (antagonist setup), depicting dose-dependent inhibition of forskolin-induced cAMP production. After pretreatment (30 min at rt) with increasing concentrations of [natLu]PentixaTher or [natLu]DOTA-r-a-ABA-iodoCPCR4, the effect of 150 ng/mL CXCL12 on cAMP reduction was measured by using the TR-FRET based LANCE assay kit. Note that [natLu]DOTA-r-a-ABA-iodoCPCR4 enhances the agonistic CXCL12-induced reduction of intracellular [cAMP](%), whereas [natLu]PentixaTher antagonizes this effect.*

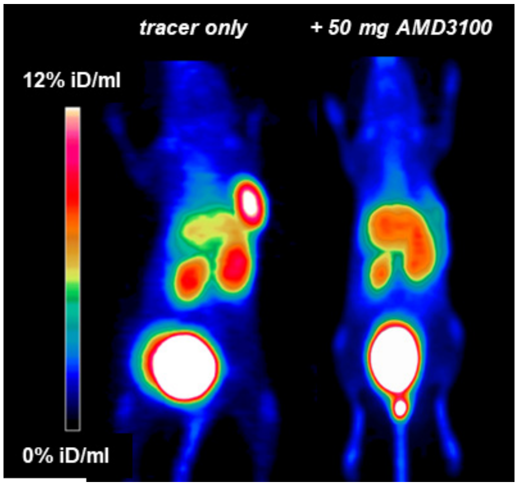

Figure S2 *[^{68}Ga]DOTA-r-a-ABA-CPCR4 PET (static imaging, 1h p.i., MIP) in Daudi xenograft bearing CB17 SCID mice; left image: tracer only, right image: coinjection of a blocking dose of 50 μg AMD3100 to confirm CXCR4-specificity of tracer accumulation in the human xenograft.*

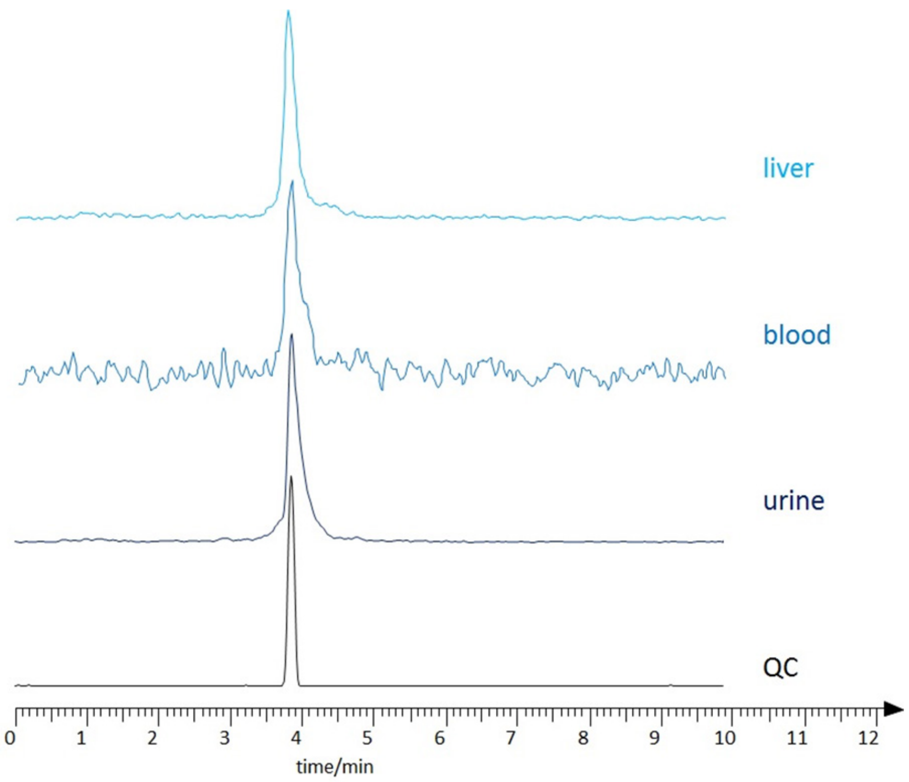

Figure S3 *In vivo metabolite analysis: exemplary radio-HPLC traces of extracts from blood, urine and liver homogenates of CB17 SCID mice 30 min p.i. of 15 MBq [^{177}Lu]DOTA-r-a-ABA-iodoCPCR4. The quality control run for the injected tracer is added for comparison.*
HPLC conditions: Chromolith Performance 18 E column, 4.6 x 100 mm; flow rate = 3 mL/min; gradient: 3 to 95% B in 6 min, UV detection at 220 nm.

References

1. Demmer O, Dijkgraaf I, Schottelius M, Wester H-J, Kessler H. Introduction of functional groups into peptides via N-alkylation. Organic letters. 2008; 10: 2015-8.
2. Demmer O, Gourni E, Schumacher U, Kessler H, Wester HJ. PET Imaging of CXCR4 Receptors in Cancer by a New Optimized Ligand. Chemmedchem. 2011; 6: 1789-91.
3. Schottelius M, Schwaiger M, Wester H-J. Rapid and high-yield solution-phase synthesis of DOTA-Tyr^3-octreotide and DOTA-Tyr^3-octreotate using unprotected DOTA. Tetrahedron letters. 2003; 44: 2393-6.
4. Schottelius M, Konrad M, Osl T, Poschenrieder A, Wester H-J. An optimized strategy for the mild and efficient solution phase iodination of tyrosine residues in bioactive peptides. Tetrahedron Lett. 2015; 56: 6602-5.
5. Weineisen M, Simecek J, Schottelius M, Schwaiger M, Wester H-J. Synthesis and preclinical evaluation of DOTAGA-conjugated PSMA ligands for functional imaging and endoradiotherapy of prostate cancer. EJNMMI Research. 2014; 1: 1-15.
6. Notni J, Pohle K, Wester H-J. Comparative gallium-68 labeling of TRAP-, NOTA-, and DOTA-peptides: practical consequences for the future of gallium-68-PET. EJNMMI research. 2012; 2: 1-5.
7. Gourni E, Demmer O, Schottelius M, D'Alessandria C, Schulz S, Dijkgraaf I, et al. PET of CXCR4 expression by a 68Ga-labeled highly specific targeted contrast agent. J Nucl Med. 2011; 52: 1803-10.
